# Theoretical Analysis on the Kinetic Isotope Effects of Bimolecular Nucleophilic Substitution (S_N_2) Reactions and Their Temperature Dependence

**DOI:** 10.3390/molecules18044816

**Published:** 2013-04-23

**Authors:** Wan-Chen Tsai, Wei-Ping Hu

**Affiliations:** Department of Chemistry and Biochemistry, National Chung Cheng University, Chia-Yi 621, Taiwan

**Keywords:** kinetic isotope effect, S_N_2 reaction, ion-molecule collision theory, transition state theory, variational transition state theory, tunneling effect

## Abstract

Factors affecting the kinetic isotope effects (KIEs) of the gas-phase S_N_2 reactions and their temperature dependence have been analyzed using the ion-molecule collision theory and the transition state theory (TST). The quantum-mechanical tunneling effects were also considered using the canonical variational theory with small curvature tunneling (CVT/SCT). We have benchmarked a few *ab initio* and density functional theory (DFT) methods for their performance in predicting the deuterium KIEs against eleven experimental values. The results showed that the MP2/aug-cc-pVDZ method gave the most accurate prediction overall. The slight inverse deuterium KIEs usually observed for the gas-phase S_N_2 reactions at room temperature were due to the balance of the normal rotational contribution and the significant inverse vibrational contribution. Since the vibrational contribution is a sensitive function of temperature while the rotation contribution is temperature independent, the KIEs are thus also temperature dependent. For S_N_2 reactions with appreciable barrier heights, the tunneling effects were predicted to contribute significantly both to the rate constants and to the carbon-13, and carbon-14 KIEs, which suggested important carbon atom tunneling at and below room temperature.

## 1. Introduction

Bimolecular nucleophilic substitution (S_N_2) reactions are ubiquitous in organic chemistry and they have been extensively studied in the last two decades [1,2,3,4,5,6,7,8,9,10,11,12,13,14,15,16,17,18,19,20,21,22,23,24,25]. Studies on the deuterium kinetic isotope effects (KIEs) have proved to be very useful for investigating the reaction mechanisms. While a methyl halide can only undergo the S_N_2 reaction pathway with a nucleophile, higher branched alkyl halides (or in general an alkane with a hydrogen substituted by an electronegative group) with β hydrogens can undergo either E2 or S_N_2 pathways [8,9,10,13,14,15,16]. It was well established that the observed deuterium KIEs could differentiate between these two pathways where the KIE at a particular temperature is defined as the ratio of the rate constant of the unsubstituted reaction to that of the deuterated reaction (k_H_/k_D_). The E2 reactions would show large “normal” deuterium KIEs (2 to 6) [13,14,15,16,26,27,28,29] at room temperature while the S_N_2 reactions would usually show slightly “inverse” KIEs (0.8 to 1.0) [12,13,14,15,16,17,18,19,20,21,22,23,24,25]. It is understood that the isotopic substitution is usually on the neutral molecule (usually an alkyl halide) and for experimental convenience, often all the hydrogens on the neutral molecule were substituted (*i.e.*, perdeuterated), even though the α-hydrogens should contribute most significantly to the S_N_2 KIEs. Nevertheless, studies with selective deuteration for the ethyl halide systems have been performed [12,15,16]. The term “normal” KIEs means that the ratios of the rate constants of the lighter isotope to that of the heavier isotope are greater than 1.0, and the “inverse” KIEs mean the ratios are less than 1.0. Many studies on the gas-phase ion-molecule reactions and their KIEs have been performed in the last two decades. For most ethyl halides, the S_N_2 pathway dominates the reaction, while for *i*-propyl halides both S_N_2 and E2 pathways might be important [8,14,15]. Most experiments were, however, done at room temperature. Since the kinetic isotope effects could well be sensitive functions of temperature, the experimental results thus only showed a small facet of the KIE trends. Furthermore, the observed KIEs require theoretical interpretation which involves considering various factors affecting the ion-molecule reaction dynamics. In the current study, we focus on the theoretical prediction and interpretation of the (deuterium, carbon-13, and carbon-14) kinetic isotope effects of the gas-phase S_N_2 reactions and their temperature dependence.

**Scheme 1:**
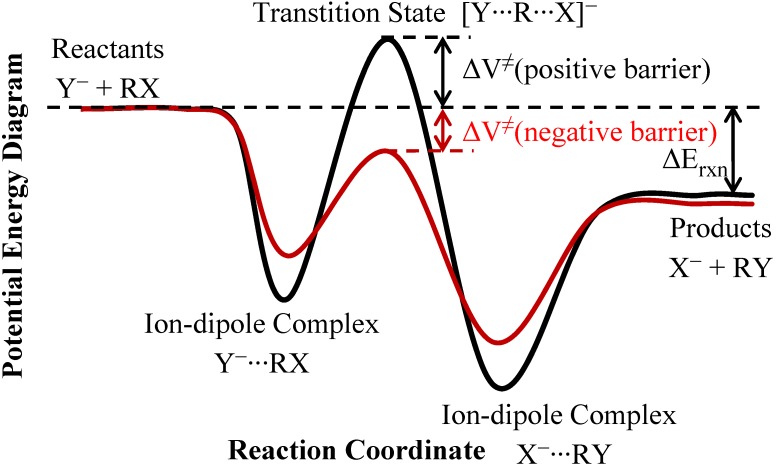
Potential energy diagram of a typical gas-phase S_N_2 reaction.

Most of the previous theoretical modeling on the S_N_2 rate constants and KIEs was based on the transition state theory (TST) [30,31,32,33,34,35,36]. However, the validity the TST modeling depends critically on the energy barriers of the reactions [13,22,37,38]. Scheme 1 shows the potential energy diagram of a typical gas-phase the S_N_2 reactions. The energy barrier of the reaction was defined as the energy of the transition state relative to the free reactants. If the barrier (in Born-Oppenheimer energy, not including the vibrational zero-point energy) is “very low” (less than approximately −7 kcal/mol), which is often the case for an exoergic ion-molecule reaction due to the strong ion-dipole interaction, the reaction bottleneck is not located at the very negative central barrier, and the bimolecular collision rates determine the overall rates and thus the KIEs almost exclusively. In such cases, the KIEs would be very close to unity since the collision rate constants, to a good approximation, are proportional to the inverse of the square root of the reduced mass of the reactants. If the TST is used alone in modeling, the predicted rate constants would be too high, and the predicted deuterium KIEs would be too low (or too inverse). For reactions with “low” barriers (approximately between −7 kcal/mol and −4 kcal/mol), both the collision process and the passage over the energy barrier of the S_N_2 reaction contribute to the reaction bottlenecks. The rate constants and KIEs can be reasonably modeled by the canonical unified statistical (CUS) theory [13,22,37,38] which takes both the collision and TST rate constants into account. In fact, the CUS theory bridges smoothly between the “very-low barrier” cases mentioned earlier, the current “low barrier” cases, and the “small barrier” cases that will be mentioned next. If the energy barrier is somewhat higher (larger than approximately −4 kcal/mol), the small barrier would usually be high enough (due primarily to the entropic effects) to form a sole bottleneck of the reaction at room temperature. The rate constants and KIEs can then be modeled quite successfully with the TST. It is noted that in the (low-pressure) gas-phase reaction, which is our focus in the current study, the barrier heights defined above determine the overall rate constants if the transition state is the sole bottleneck. The energies of the ion-dipole complexes, which are usually 10–20 kcal/mol lower than those of the reactants and products (see Scheme 1), do not affect the overall reaction rates. In the condensed phase or at high pressure where the low-energy complexes could be stabilized before dissociation, the energy difference between the transition state and the reactant-side complex would become an important factor determining the rate constants. However, in polar solvent, the solvation energies of the reactants are usually significantly larger than the solvation energies of the TS and of the ion-dipole complexes, and the energy wells of the ion-dipole complexes would become much shallower.

Quantitatively agreement with experimental rate constants and deuterium KIEs have been made in a few systems by theoretical prediction based on TST [4,16,17,21,22]. However, different electronic structure theories usually give different prediction on the values of KIEs [21,22,24,29]. There is no consensus on which theoretical methods are more reliable than the others. Currently, the rates of high-barrier (approximately higher than 3 kcal/mol or with rate constants lower than 10^−^^12 ^cm^3^·molecule^−^^1^·s^−^^1^) gas-phase S_N_2 reactions are too slow to be measured reliably. It is expected, however, the quantum mechanical tunneling effects may play an important role in determining the rate constants at lower temperature since the widths of the barriers of S_N_2 reactions tend to be narrow due to the deep energy wells of the ion-dipole complexes before and after the central energy barriers along the reaction paths. To model the rate constants and the KIEs correctly for the “high-barrier” cases, the tunneling effects need to be calculated accurately, which is understandably a very challenging task. In the current work, we will investigate the theoretical methods that are suitable to model the KIEs of the S_N_2 reactions with a large range of barrier heights and at a wide range of temperature. Suitable electronic structure methods will be chosen from a benchmark study against suitable experimental values for the prediction of deuterium KIEs using TST for the “small barrier” cases. Tunneling contribution to the deuterium, ^13^C-, and ^14^C-KIEs of the S_N_2 reactions will be modeled for the reaction of CN^−^ + CH_3_OCl in the gas phase.

## 2. Theory and Methods

### 2.1. KIE Definition

The kinetic isotope effect at a particular temperature is defined as the ratio the rate constant of the unsubstituted reaction to that of the isotopically substituted reaction. Alternatively, it can be more generally defined as the ratio of the rate constants with lighter isotope to that with heavier isotope:

KIE = *k*^light^/*k*^heavy^ (1)

For example, the deuterium and carbon-13 KIEs are defined as *k*_H_/*k*_D_ and *k*12_C_/*k*13_C_, respectively. 

### 2.2. KIEs Calculation from the Capture Rate Constants

In the “very low barrier” cases, the ion-molecule S_N_2 reaction can be modeled by a simple ion-dipole capture process. The capture rate constant can be expressed as:

*k*^cap^ = *c*(*T*) *k*_*L*_ (2)

*k*_*L*_ = 2π*q*(α/*m*)^1/2^ (3)

where *c* is a function of temperature, *k_L_* is the Langevin-Gioumousis-Stevenson rate constants [39], *q* is the charge of the ion, α is the polarizability of the neutral molecule, and *m* is the reduced mass defined by:

*m* = *m*_*i*_*m*_*d*_/(*m*_*i*_ + *m*_*d*_) (4)

where *m_i_* and *m_d_* are the mass of the ion and the dipole (the neutral molecule), respectively. The factors *c* and α, to a good approximation, are assumed to be independent of the isotopic mass. For example, Celli *et al*. [40] proposed a convenient formula for the capture rate constants:

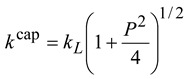
(5)

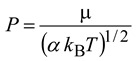
(6)
where μ is the dipole moment of the neutral molecule, *k*_B_ is the Boltzmann constant, and *T* is the absolute temperature. A similar but more sophisticated method developed by Chesnavich *et al*. was also often used [39]. The KIE of the capture process would thus be: 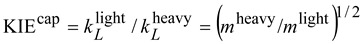
(7)

If the isotope substitution is localized on the neutral molecule, which is often the case, the KIE can be expressed as:
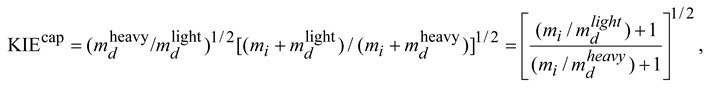
(8)
which is usually only slightly larger than unity. Thus if the reaction bottleneck is at the collision process, then the KIEs are almost negligible. The capture rate constants can also be used to estimate the reaction efficiency:

*f* = *k*^expt^ / *k*^cap^ (9)

where *k*expt is the experimental S_N_2 rate constant.

### 2.3. KIEs Calculation Using the Canonical Unified Statistical (CUS) Model

In the “low barrier” cases, both the ion-molecule collision and the passage of the energy barrier contribute to the reaction bottleneck, and the S_N_2 rate constants can be adequately modeled by the CUS method [13,22,37,38]:
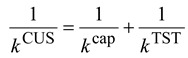
(10)
The KIEs calculated by the CUS model can be written as:
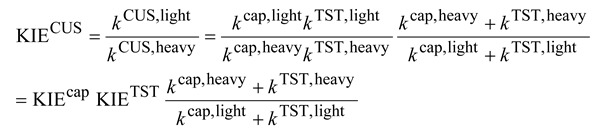
(11)
where the KIE^cap^ can be obtained from Equation (7) above, and the *k*^TST^ and KIE^TST^ are the rate constants and KIE calculated from transition state theory which will be described below. It is easily seen that the last term in Equation (11) becomes the inverse of KIEcap if *k*^cap^ >> *k*^TST^ (“small barrier” case) and becomes the inverse of KIE^TST^ if *k*^cap^ << *k*^TST^ (“very low barrier” case). Thus the KIE^CUS^ would normally be between the two extremes and be slight inverse, but higher than KIE^TST^.

### 2.4. KIEs Calculation Using the Transition State Theory (TST)

The rate constant predicted by the transition state theory can be written as:
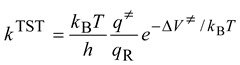
(12)
where *h* is the Planck constant, *q≠* and *q*R are partition functions per unit volume of the transition state and the reactants, respectively, and *∆V* *≠* is the barrier height. In Equation (12), the vibrational zero-point energies are included in the vibrational partition functions and *∆V* *≠* includes only Born-Oppenheimer energies. For reactions with small barrier heights such that *k*^cap^>> *k*^TST^, the rate constants and thus the KIEs are adequately modeled by the TST. Within the Born-Oppenheimer approximation, the isotopic substitution does not affect the barrier height. Thus the KIEs only involve the ratios of the partition functions which can be factorized into translational, rotational, and vibrational contributions [17,21,22,24,41,42,43]. Using the deuterium KIE as an example:
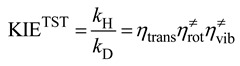
(13)

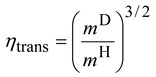
(14)

If the isotope substitution is exclusively on the neutral molecule, from Equation (4) the above equation can be rewritten as:
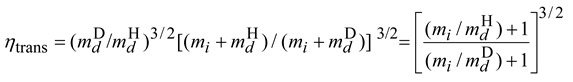
(14’)

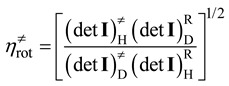
(15)

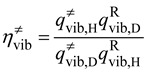
(16)

where *m*^H^ and *m*^D^ are the reduced masses (see Equation (4) above) of the unsubstituted and deuterated systems, respectively, det I is the determinant of moment of inertia, *q*_vib_ is the vibrational partition function. Superscripts of “R” and “≠” signify the quantities are evaluated at the reactants and at the transition state, respectively. All these quantities can in principle be calculated with electronic structure calculation (that is, geometry optimization followed by vibrational frequency calculation.) The harmonic approximation is normally used to calculate the vibrational partition functions. The translation contribution is usually slightly normal due to the relatively small difference on the molecular masses between the unsubstituted and deuterated systems. The rotational contribution is usually significantly normal with values around 1.2–1.6 for smaller alkyl halides. This is mainly because in the transition state, the α-hydrogens are in more extended positions, which makes the det I of the deuterated transition state significantly larger. It is noted that both the translational and rotational contributions are temperature independent. The temperature dependence of KIE^TST^ thus comes exclusively from the vibrational contribution. The vibrational contribution to the deuterium KIE is usually inverse, and it is normally 0.5–0.8 at 300 K. The general rule is that the more inverse the vibrational contribution is, the stronger temperature dependence it shows. While traditionally large KIEs were often attributed to the differences in vibrational zero-point energies (ZPE) between the transition state and the reactants. For the gas-phase S_N_2 reactions, however, the ZPE differences are usually not very significant. Instead, as will be shown below, the inverse vibrational contribution arises from the combined effects of various types of vibrational modes. That is, the slightly inverse deuterium KIEs are due to the overall “entropic” effects, not the “energetic” effects. In cases where both the capture and TST rate constants need to be considered to calculate the KIEs, as in the “low barrier” cases, then Equation (11) can be rewritten as:
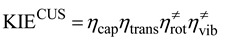
(17)


The variational effects, which signify the shifting the bottleneck away from the transition state, are usually very small for S_N_2 reactions due to the relatively sharp shape of the energy barrier. Thus in the current work the discussion on the variational effects are neglected. However, the variational effects are nonetheless included in the calculation when the tunneling corrections are applied.

### 2.5. KIEs Calculation with Tunneling Correction

The S_N_2 reaction paths involve primarily the movement of heavy atoms. Thus it was not expected that the tunneling effects would be very important. However, recent studies showed that carbon atom tunneling could be significant at and below room temperature for reactions with a narrow and appreciable barrier [25,44,45,46,47,48,49,50]. The tunneling correction to the calculated rate constant is usually applied by multiplying the TST rate constants with a temperature-dependent correcting factor [36,42,43,51,52]:

*k*^TST/Tunneling^ = κ^tunneling^*k*^TST^ (18)

One of the popular and simplest methods to obtain the κ factor is by the Wigner tunneling formula [51,52]:
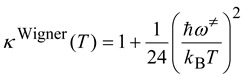
(19)
where the *ω*≠is the imaginary vibrational frequency of the transition state. However, obtaining accurate estimation of the tunneling effects requires the information of the potential energy surface along the full reaction path. The most successful semi-classical tunneling theory used with the TST is the small-curvature tunneling (SCT) method [36,43,53,54] which not only considers the whole reaction-path topology but also takes the reaction-path curvature into consideration. The calculated rate constants with the SCT correction are called *k*^TST/SCT^. If the variational effects are considered using the popular canonical variational theory (CVT) [35,36] which locates a single reaction bottleneck at a particular temperature along the reaction path, the resulting rate constants are called *k*CVT/SCT [17,43,53,54,55,56]:

*k*^TST/SCT^ = κ^SCT^*k*^TST^ (20)

*k*^CVT/SCT^ = κ^SCT^*k*^CVT^ (21)

The use of Equation (21) is more logical since the reaction path has been calculated to obtain the κ^SCT^. The tunneling contribution (neglecting the variational contribution) to the KIEs can be written as (using deuterium KIE as an example):
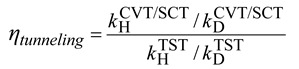
(22)
and Equation (13) can be rewritten as:

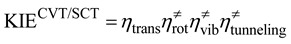
(23)

The tunneling effects are usually expected to make a normal (>1.0) contribution to the KIEs since the lighter isotope usually involves more significant tunneling. However, at low temperature, the deuterium KIEs predicted by TST can sometimes be too large for a reaction with a significant normal KIE at room temperature, for example in typical E2 reactions. If the reaction is dominated by tunneling effects, then the predicted KIEs by CVT/SCT theory could be significantly smaller since the tunneling effects are less sensitive to the barrier heights. In such cases, the tunneling contribution to the deuterium KIEs would be inverse to prevent the KIEs from becoming infinite as temperature approaches absolute zero [26]. The tunneling contribution for heavy-atoms (^13^C, ^14^C, ^18^O, *etc*.) KIEs is usually normal but smaller in magnitude due to the less significant tunneling effects. However, in S_N_2 reactions, the reaction path directly involves the motion of the α-carbon atom, thus the tunneling contribution to the carbon KIEs can be more significant than to the deuterium KIEs.

### 2.6. Electronic Structure Calculation

To benchmark the performance of the electronic structure methods in predicting the deuterium KIEs (using the transition state theory) against experiment values, the molecular geometry and vibrational frequencies of the reactants and transition states of 11 S_N_2 reactions were calculated using the B3LYP [57] and M06-2X [58] hybrid functional and the MP2 [59] theory with the 6-31+G(d,p) [60,61,62], 6-311+G(d,p) [62,63,64], aug-cc-pVDZ, and aug-cc-pVTZ [65,66,67] basis sets. Additionally, geometry and frequency calculation at the CCSD(T) [68]/aug-cc-pVTZ level was also performed for the Cl^−^ + CH_3_I, CN^−^ + CH_3_I, and Cl^−^ + CH_3_Br reactions. Furthermore, anharmonic frequencies and anharmonic vibrational-rotational couplings [69,70,71,72] were also calculated for the Br^−^ + CH_3_I and Cl^−^ + CH_3_Br reactions. For the iodine atom, the aug-cc-pVDZ-pp [73] basis set was used with the 6-31+G(d,p) and aug-cc-pVDZ basis sets for other atoms, and the aug-cc-pVTZ-pp [73] basis set was used with the aug-cc-pVTZ basis set for other atoms. For the chlorine and sulfur atoms, the aug-cc-pV(D+d)Z and aug-cc-pV(T+d)Z [74] basis sets were used with the aug-cc-pVDZ and aug-cc-pVTZ basis sets, respectively, for other atoms.

For the CN^−^ + CH_3_OCl reaction, which we chose for studying the tunneling effect and tunneling contribution to the KIEs, the structures and vibrational frequencies were calculated using the B3LYP hybrid functional with the 6-311+G(d,p) basis set and the MP2 theory with aug-cc-pVDZ and aug-cc-pVTZ basis sets. (For the chlorine atom, the aug-cc-pV(D+d)Z and aug-cc-pV(T+d)Z basis sets were used.) Single-point calculations for reaction energy and barrier height were calculated at the CCSD(T)/aug-cc-pVTZ level. All electronic structure calculations and anharmonic analysis were performed using the Gaussian 09 program [75].

### 2.7. Rate Constant and KIEs Calculation

The ion-molecule capture rate constants were calculated using Celli *et al*.’s method, as shown in Equation (5), and the experimental dipole moment and polarizability was obtained from the experimental values [76]. The KIE^cap^ were calculated directly from Equation (8). The CUS rate constants were calculated using the Equation (10), and the KIE^CUS^ using Equation (11). The TST rate constants were obtained using the Equation (12), and the information needed to calculate the partition functions were obtained from the electronic structure calculation at various levels of theory, and the harmonic approximation was used for vibration. The KIE^TST^ and its various contributions were calculated by Equations (13)–(16). For the CN^−^ + CH_3_OCl reaction and its various isotopically substituted analogs, the rate constants were calculated using the dual-level [77,78] variational transition state theory with multidimensional tunneling (VTST/MT) [35,36,53,54,55] correction. The dual-level method requires a qualitatively correct “low-level” potential energy surface (PES), and a set of “high-level” energy data on the stationary points along the reaction path for the interpolated corrections to the low-level PES. The low-level PES for the VTST/MT calculation was obtained by using the MP2/aug-cc-pVDZ (aug-cc-pV(D+d)Z for the chloride atom) method. The reaction path was calculated using the Page-McIver method [79,80] from −2.5 to 2.5 bohrs with the gradient and hessian step sizes of 0.006 and 0.03 bohrs, respectively in the mass-scaled coordinates with a scaling mass of 1 amu. Redundant internal coordinate systems [81,82,83,84] were used in the vibrational analysis along the reaction path. The high-level energies were obtained at the CCSD(T)/aug-cc-pVTZ level, and the interpolated correction was based on the ion-dipole complexes. The SIL-1 interpolated correction scheme [85] was applied in the dual-level calculations using the CCSD(T)/aug-cc-pVTZ energies along the low-level reaction paths to estimate the accurate barrier widths. The rate constants were calculated from 50 K to 600 K at the conventional transitional state theory (TST), canonical variational theory (CVT), and canonical variational theory with tunneling correction at the small-curvature tunneling [43,53,54,55] level (CVT/SCT). The overall symmetry number was set to one. The dual-level VTST/MT calculations were performed using the GAUSSRATE 8.4H program, which is a locally modified version of the GAUSSRATE 8.2 program [86], and it served as an interface between the GGAUSSIAN 09 and the POLYRATE 8.2 programs [87].

## 3. Results and Discussion

### 3.1. Systems with Very Low Barriers (Collision-Controlled Reactions)

Table 1 shows the experimental and calculated capture rate constants and comparison of the experimental and predicted deuterium KIEs (perdeuterated alkyl halides) for some very fast gas-phase S_N_2 reactions with bimolecular rate constant around 10^−9^ cm^3^·molecule^−1^·s^−1^. The reaction bottlenecks of these systems are apparently in the ion-molecule collision rates. As seen in the table, the observed deuterium KIEs were all very close to unity, which are consistent with the KIE^cap^ calculated from Equation (8). An important point here is that for very fast S_N_2 reactions, the observed KIEs do not reflect the properties of the transition states involved or the reaction paths on which the systems follow, since in such cases the bottlenecks are located very early in the entrance channels. That is, a deuterium KIE very close to unity, for example in the case of H_2_NS^−^ + CH_3_CH_2_Br, does not necessarily mean that the system takes the S_N_2 pathway. In such cases, the temperature dependence of the KIEs is expected to be the negligible, as predicted from Equations (2)–(8).

**Table 1:** Experimental and calculated capture rate constants (in cm^3^ molecule^−1^ s^−1^) and deuterium KIEs of some very fast S_N_2 reactions at room temperature in the gas phase.

	***k*^cap^**	***k*^expt^**	**Efficiency**	**KIE^cap^**	**KIE^expt^**
F^−^ + CH_3_Br ^*a*^	2.97(−09) ^*c*^	1.88(−09)	0.631	1.003	0.98 ± 0.02
F^−^ + CH_3_I ^*a*^	2.79(−09)	1.94(−09)	0.695	1.001	0.98 ± 0.05
CF_3_CH_2_O^−^ + CH_3_CH_2_Br ^*b*^	1.87(−09)	1.24(−09)	0.665	1.011	1.10 ± 0.06
CF_3_CH_2_O^−^ + (CH_3_)_2_CHBr ^*b*^	1.98(−09)	1.39(−09)	0.703	1.012	1.20 ± 0.05
H_2_NS^−^ + CH_3_Br ^*b*^	2.10(−09)	7.04(−10)	0.336	1.005	1.04 ± 0.03
H_2_NS^−^ + CH_3_CH_2_Br ^*b*^	2.33(−09)	9.05(−10)	0.389	1.007	1.00 ± 0.04
CF_3_CF_2_CH_2_O^−^ + CH_3_Br ^*b*^	1.56(−09)	8.48(−10)	0.545	1.010	0.99 ± 0.04
CF_3_CF_2_CH_2_O^−^ + CH_3_CH_2_Br ^*b*^	1.69(−09)	9.86(−10)	0.582	1.013	1.16 ± 0.06

^*a*^ Experimental values from ref. [20] at 302 K; ^*b*^ Experimental values from reference [8], calculations done at 300 K; ^*c*^ 2.97(−09) means 2.97 × 10^−9^.

### 3.2. Systems with Low Barriers (both Collision- and TS-Controlled Reactions)

As mentioned in the Theory and Methods section, when the energy barrier along the S_N_2 reaction path is appreciable such that the calculated TST rate constants are comparable to the capture rate constants, both the ion-molecule collisions and the passage over the TS contribute to the reaction bottleneck. In such cases, the CUS theory [Equation (10)] provided a reasonable model and continuous interpolation between the very-low barrier, collision controlled cases to the small barrier, transition-state controlled cases. However, there are practical difficulties in applying Equations (9) and (10). While the collision rate constants can be estimated quite easily and different models gave similar (within ~10%) results [13,39,40], the calculated TST rate constants are subject to much larger uncertainty due mainly to the uncertainty on the predicted barrier heights. A mere 2 kcal/mol error in the calculated barrier height would cause an error on the TST rate constant by a factor of almost 30 at 300 K. Recent benchmark study showed that the most accurate DFT and *ab initio* methods can consistently predict barrier heights of heavy atom transfer reaction to around 1 kcal/mol and 0.5 kcal/mol, respectively [58,88,89,90,91]. This would still cause significant uncertainty (at least by a factor of two) on the calculated TST rate constants. As a result, accurate prediction of KIE^CUS^ by Equation (11) is difficult. This situation is illustrated in Table 2. 

**Table 2:** Calculated reaction energetics (in kcal/mol), the experimental and theoretical rate constants (in cm^3^ molecule^−1^ s^−1^), and KIEs of the F^−^(H_2_O) + CH_3_Br reaction at 302 K in the gas phase at various levels of theory.

	**F^−^(H_2_O) + CH_3_Br/F^−^(H_2_O) + CD_3_Br ^*a*^**					
	**∆V^≠^**	**∆E_rxn_**	***k*^TST^**	***k*^CUS^**	**KIE^TST^**	**KIE^CUS^**
M06-2X/6-31+G(d,p)	−7.8	−28.2	4.11(−09) ^*b*^	1.47(−09)	0.795	0.929
M06-2X/6-311+G(d,p)	−8.6	−30.4	2.87(−08)	2.12(−09)	0.628	0.977
M06-2X/aug-cc-pVDZ	−8.9	−31.0	1.40(−08)	1.97(−09)	0.763	0.970
B3LYP/6-31+G(d,p)	−9.7	−23.8	1.39(−06)	2.29(−09)	0.832	1.004
B3LYP/6-311+G(d,p)	−10.5	−25.7	2.76(−05)	2.29(−09)	0.836	1.004
MP2/6-31+G(d,p)	−5.8	−23.6	7.38(−09)	1.75(−09)	0.868	0.972
MP2/6-311+G(d,p)	−2.2	−23.2	4.02(−11)	3.95(−11)	0.833	0.836
MP2/aug-cc-pVDZ	−6.3	−22.8	1.48(−09)	8.97(−10)	0.820	0.892
MP2/aug-cc-pVTZ	−4.0	−21.7	6.61(−11)	6.43(−11)	0.857	0.861
CCSD(T)/aug-cc-pVTZ ^*c*^	−6.6	−24.0	2.52(−09)	1.20(−09)	0.820	0.917

^*a*^ Experimental rate constant and KIE of 4.97 × 10^−9^ cm^3^·molecule^−1^·s^−1 ^ and 0.92, respectively, are from ref. [20] at 302 K. Calculated capture rate constant and KIE are 2.29 × 10^−9^ cm^3^·molecule^−1^·s^−1^ and 1.004, respectively; ^*b*^ 4.11(−09) means 4.11 × 10^−9^; ^*c*^ Using the geometries and frequencies calculated at the MP2/aug-cc-pVDZ level.

For the S_N_2 reaction of F^−^(H_2_O) + CH_3_Br, different electronic structure methods predicted significantly different barrier heights. As seen in the table they range from −2.2 kcal/mol by MP2/6-311+G(d,p) to −10.5 kcal/mol by B3LYP/6-311+G(d,p). The predicted deuterium KIE ranged from 0.84 (totally TS controlled) to 1.00 (totally collision controlled). However, if we further calculate the single-point energies at the CCSD(T)/aug-cc-pVTZ//MP2/aug-cc-pVDZ level, we obtained a barrier height of −6.6 kcal/mol. Using this much more accurate barrier heights and taking the geometry and frequencies calculated at the MP2/aug-cc-pVDZ level, we obtained a KIE^CUS^ of 0.92 which is in good agreement with the experimental result (0.92 ± 0.03) even though the predicted *k*^CUS^ rate constant was still a factor of 2.5 higher than the experimental value [20]. The obtained KIE^CUS^ value (0.92) is compared to the predicted KIE^TST^ and KIE^cap^ values of 0.82 and 1.00, respectively. This clearly shows once again that the CUS model gives a valid interpolation on the KIE between the TST and ion-molecule collision model for systems with low barriers.

### 3.3. Systems with Small Barriers (TS Controlled Reactions)

When the energy barrier is above approximately −4 kcal/mol, or the experimental rate constant is below approximately 2 × 10^−10^ cm^3^·molecule^−1^·s^−1^ (reaction efficiency < 10%), the TST rate constant at 300 K would be significantly lower than the capture rate constant. In such cases, the reaction bottlenecks are located on the passage over the TS, and the rate constants, and thus the KIEs, can be adequately modeled by the transition state theory. Since the molecular geometry and energy barriers are, within Born-Oppenheimer approximation, independent of the isotopic substitutions, the KIEs calculated by TST can be factored into simple ratios of partition functions calculated at the reactants and the transition state, as shown in Equations (13)–(16). However, some of these ratios are sensitive to the choices of the methods employed in the electronic structure calculation.

#### 3.3.1. Benchmark of Electronic Structure Methods

In the current study we benchmark the performance of MP2, B3LYP, and M06-2X theory with a few commonly used basis sets against the experimental deuterium KIEs of eleven gas-phase S_N_2 systems where in the deuterated reactions all the hydrogens in the neutral molecules are deuterated. The properties of these reactions are listed in Table 3. The reaction efficiencies of all the reactions were estimated to be below 10%, and the E2 contributions to the four ethyl halide systems were assumed to be negligible. (The zero-point corrected barriers of E2 channels were predicted to be at least 3 kcal/mol higher at the CCSD(T)/aug-cc-pVTZ level. See the Supplementary Material.) The KIE^expt^ in most cases were significantly different from the KIEcap, meaning that the reaction bottlenecks were not at the collision processes.

**Table 3:** Experimental and calculated capture rate constants (in cm^3^ molecule^−1^ s^−1^) and deuterium KIEs of eleven gas-phase S_N_2 reactions with reaction efficiencies < 10% at room temperature.

	***k*cap**	***k*expt**	**Efficiency**	**KIEcap**	**KIEexpt**
ClO^−^ + CH_3_Cl *^a^*	2.37(−09) *^f^*	2.01(−10)	0.085	1.015	0.85 ± 0.01
ClO^−^ + CH_3_CH_2_Cl *^a^*	2.46(−09)	2.25(−10)	0.091	1.016	0.99 ± 0.01
BrO^−^ + CH_3_Cl *^a^*	2.08(−09)	1.08(−10)	0.052	1.019	0.82 ± 0.03
BrO^−^ + CH_3_CH_2_Cl *^a^*	2.12(−09)	1.07(−10)	0.050	1.022	0.96 ± 0.03
HS^−^ + CH_3_CH_2_Br *^b^*	2.67(−09)	1.95(−10)	0.073	1.005	1.02 ± 0.07
Cl^−^ + CH_3_I *^b^*	2.15(−09)	1.66(−10)	0.077	1.002	0.84 ± 0.02
Br^−^ + CH_3_I *^b^*	1.60(−09)	2.89(−11)	0.018	1.004	0.76 ± 0.03
CN^−^ + CH_3_I *^c^*	2.44(−09)	1.28(−10)	0.052	1.002	0.84 ± 0.03
CN^−^ + CH_3_CH_2_I *^c^*	2.81(−09)	2.99(−11)	0.011	1.002	0.89 ± 0.02
Cl^−^ + CH_3_Br *^d^*	2.33(−09)	2.37(−11)	0.010	1.004	0.88 ± 0.45
F^−^(H_2_O) + CH_3_Cl *^e^*	2.59(−09)	1.49(−11)	0.006	1.012	0.85 ± 0.03

*^a^* Experimental values from ref. [15] at 302 K; *^b^* Experimental values from ref. [8], calculations done at 300 K; *^c^* Experimental values from ref. [16] at 298 K; *^d^* Experimental values from ref. [18] at 300 K; *^e^* Experimental values from ref. [20] at 302 K; *^f^* 2.37(−09) means 2.37 × 10−9.

The performance of the tested methods is summarized in Table 4. The overall best methods in reproducing the experimental results are MP2/aug-cc-pVDZ and MP2/6-31+G(d,p) with mean unsigned errors (MUE) of 0.049 and 0.068, respectively. The detailed test results were included in the See the supplementary material. The best DFT method is M06-2X/aug-cc-pVDZ with an MUE of 0.079. The popular B3LYP/6-31+G(d,p) method gave an MUE 163% larger than that of the MP2/aug-cc-pVDZ method. It is noted that in most cases, the predicted KIEs are higher than the experimental values by a few percent to twenty percent. The origins of the almost systematic discrepancies are still unclear. For two of the reactions: CN^−^ + CH_3_I and Cl^−^ + CH_3_Br, we tested the high-level CCSD(T)/aug-cc-pVTZ method, and the obtained KIEs were still similar to the MP2/aug-cc-pVTZ results. We also tested simple anharmonic treatment of the vibrations and vibration-rotation coupling for Br^−^ + CH_3_I and Cl^−^ + CH_3_Br systems, but the resulting KIEs were either higher or similar to the values obtained with the harmonic treatment. This result is consistent with an earlier study [22] that including anharmonicity did not reduce the disagreement with experiment. Future experimental and theoretical studies on this respect are desired to resolve the discrepancies between theory and experiments, especially for the Br^–^ + CH_3_I system which has a very inverse experimental deuterium KIE of 0.76.

**Table 4:** Mean unsigned errors (MUE) *^a^* and standard deviations (SD) on the KIEs of the eleven S_N_2 reactions at various levels of theory.

	**MUE**	**SD**
M06-2X/6-31+G(d,p)	0.153	0.231
M06-2X/6-311+G(d,p)	0.087	0.055
M06-2X/aug-cc-pVDZ	0.079	0.058
B3LYP/6-31+G(d,p)	0.129	0.070
B3LYP/6-311+G(d,p)	0.172	0.094
B3LYP/aug-cc-pVDZ	0.099	0.056
MP2/6-31+G(d,p)	0.068	0.038
MP2/6-311+G(d,p)	0.121	0.073
MP2/aug-cc-pVDZ	0.049	0.039
MP2/aug-cc-pVTZ	0.075	0.047

*^a^* MUE was defined as the average of the unsigned differences between the calculated and experimental KIEs.

#### 3.3.2. Factor Analysis of KIE

According to Equations (12)–(16), the KIE can be factored into contributions from the translational, rotational, and vibrational motions. Table 5 listed these contributions to the deuterium KIEs at 300 K calculated at the MP2/aug-cc-pVDZ level for the eleven reactions mentioned above and two additional reactions: CN^−^ + (CH_3_)_2_CHI, and CN^−^ + (CH_3_)_3_CI where in the deuterated reactions all the hydrogens on the neutral molecules were assumed to be deuterated.

#### 3.3.2.1. Translational Contribution

The translational contribution are only slightly normal, and according to Equation (14’) is larger for heavier anions. For example, as seen in Table 5 the translational contribution was 1.006 for the Cl^−^ + CH_3_I system and was 1.069 for the BrO^−^ + CH_3_CH_2_Cl system.

#### 3.3.2.2. Rotational Contribution

The rotational contribution derived from the ratios of moments of inertia, as seen in Equation (15), and is usually significantly larger than the translational contribution. It is largest in the systems containing methyl halides and molecular anions, such as BrO^−^, that contain heavy atoms not bonded directly to the primary carbon in the product. In such systems, one of the principle axes is moved away from the primary carbon in the transition state, which magnifies the substitution effects on the rotational contribution. As seen in Table 5, the rotational contributions for ClO^−^ + CH_3_Cl and BrO^−^ + CH_3_Cl were 1.617 and 1.625, respectively. The microsolvated reaction of F^−^(H_2_O) + CH_3_Cl also shows a large rotational contribution of 1.660 for similar geometrical reasons. All other systems showed rotational contributions of 1.2–1.3. For example, the rotational contributions for Cl^−^ + CH_3_I and Br^−^ + CH_3_I were 1.231 and 1.241, respectively.

#### 3.3.2.3. Vibrational Contribution

The vibrational contribution is very often the dominant factor in determining the kinetic isotope effects. It involves, as shown in Equation (16), all the vibrational modes of the transition states and the reactants for both the isotopically substituted and unsubstituted systems. It has been shown that the inverse vibrational contribution to the deuterium KIEs was due both to the high- and low-frequency modes [12,13,17,21,22,24,92,93,94,95]. The high-frequency contribution derives from the higher C_α_-H stretching frequencies at the TS than at the reactant. The C_α_-H bonds apparently are strengthened from the reactant to the TS with the bond lengths shortened by ~0.01 Å [22]. The lowest-frequency or the “transitional” modes are intrinsically “inverse” contributors to the deuterium KIEs. These modes are present only at the transition state, and the frequencies of these modes in the unsubstituted systems are almost always higher than those in the deuterated system. Thus these transitional modes would also make inverse contribution to the deuterium KIEs. The mid-frequency modes usually contribute somewhat normally to the deuterium KIEs. The overall vibrational contributions are usually in the range of 0.5–0.8. For example, in the Cl^−^ + CH_3_Br system, the calculated vibrational contribution at 300 K is 0.732 which is then multiplied by the translational contribution (1.013) and rotational contribution (1.234) to give the predicted KIE of 0.915. For ethyl halides, there are one fewer α-hydrogens, and thus the vibrational contribution is expected to be less inverse. As shown in Table 5, the vibrational contributions for the ClO^−^ + CH_3_Cl and ClO^−^ + CH_3_CH_2_Cl systems were 0.527 and 0.717, respectively. Similar trends were observed if the anion was replaced by BrO^−^. It is worth mentioning that the recent selective-deuteration study by Bierbaum, Westaway, and coworkers [15,16] clearly showed that the β-D3 KIEs of CH_3_CH_2_Cl + ClO^−^/BrO^−^ and CH_3_CH_2_I + CN^−^ were slightly normal while the α-D2 and perdeutero (D5) KIEs were slightly inverse. These results confirmed that the inverse perdeutero KIEs which were usually observed in the gas-phase S_N_2 reactions were caused solely by the deuteration of the α-hydrogens. Table 5 suggested that the larger value of the vibrational contribution in the HS^−^ + CH_3_CH_2_Br system (0.831) makes the deuterium KIE normal. This is consistent with the experimental normal KIE of 1.02. However, the trends were less pronounced for the CN^−^ + CH_3_I / CH_3_CH_2_I / (CH_3_)_2_CHI series. For the *t*-butyl halides, the E2 pathway is expected to be dominant [16]. Nonetheless, it is noted that theoretically the deuterium KIEs of the *t*-butyl halides in the S_N_2 channel do not necessarily become close to unity or less inverse than the ethyl or the *i*-propyl halides. This is because in such systems neither the rotational nor the vibrational contributions have to be very close to unity, and that the greater number of deuterated β-hydrogens can also make significant inverse contribution, as shown in Table 5 for the CN^−^ + (CH_3_)_3_CI / (CD_3_)_3_CI system. It is also worth mentioning that experimentally if the measured deuterium KIE of an ethyl halide system is significantly higher than that of a methyl halide or is slightly larger than 1.0, this does not necessarily mean that the E2 channel also contributes to the KIE [15]. As seen from the example mentioned above, the relative masses (translational contribution), the molecular geometry of the TS, and the changes in vibrational frequencies from the reactants to the TS, all contribute to the deuterium KIEs. This often made the interpretation of observed trends in KIEs difficult without accurate theoretical modeling. It is also noted that for the reactions listed in Table 3, except for F^−^(H_2_O) + CH_3_Cl, the vibrational zero-point energies of the reactants and the transition state are all very similar (see Table S5 in the supplementary material). As mentioned in Section 2.4, the inverse vibrational contribution in gas-phase S_N_2 reactions was *not* due to the overall zero-point energy effects.

**Table 5:** Calculated translational, rotational, and vibrational contributions to the deuterium KIEs at 300 K.

	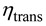	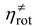	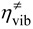	**KIE^TST^*a***	**KIEexpt *b***
ClO^−^ + CH_3_Cl	1.045	1.617	0.527	0.890	0.85 ± 0.01
ClO^−^ + CH_3_CH_2_Cl	1.050	1.310	0.717	0.987	0.99 ± 0.01
BrO^−^ + CH_3_Cl	1.059	1.625	0.519	0.893	0.82 ± 0.03
BrO^−^ + CH_3_CH_2_Cl	1.069	1.312	0.710	0.995	0.96 ± 0.03
HS^−^ + CH_3_CH_2_Br	1.016	1.271	0.831	1.073	1.02 ± 0.07
Cl^−^ + CH_3_I	1.006	1.231	0.717	0.889	0.84 ± 0.02
Br^−^ + CH_3_I	1.011	1.241	0.731	0.918	0.76 ± 0.03
CN^−^ + CH_3_I	1.005	1.229	0.714	0.881	0.84 ± 0.03
CN^−^ + CH_3_CH_2_I	1.007	1.264	0.724	0.921	0.89 ± 0.02
Cl^−^ + CH_3_Br	1.013	1.234	0.732	0.915	0.88 ± 0.45
F^−^(H_2_O) + CH_3_Cl	1.037	1.660	0.481	0.828	0.85 ± 0.03
CN^−^ + (CH_3_)_2_CHI	1.008	1.170	0.787	0.928	
CN^−^ + (CH_3_)_3_CI	1.009	1.129	0.767	0.874	

*^a^* KIEs predicted by the transition state theory; *^b^* Experimental KIEs, see Table 3.

#### 3.3.3. Temperature Dependence of the KIEs

The vibrational contribution is the main source of the temperature dependence of the KIEs for the systems where the application of the TST theory is valid. The calculated temperature dependence of the KIE and its vibrational contribution for several reactions was listed in Table 6. For example, the vibrational contribution of the Cl^−^ + CH_3_Br system from 200 K to 600 K was predicted to increase from 0.695 to 0.749 and the corresponding KIE from 0.868 to 0.937. Similarly, the vibrational contribution of the F^−^(H_2_O) + CH_3_Cl system from 200 K to 600 K was predicted to increase from 0.425 to 0.527 and the corresponding KIE from 0.732 to 0.907. It would be very interesting to compare the predicted temperature dependence with future experiments which can measure KIEs at different temperature. For systems with only small vibrational contribution (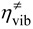 close to 1.00), the temperature dependence is expected to be small. For example, the ClO^−^ + CH_3_CH_2_Cl and BrO^−^ + CH_3_CH_2_Cl systems were predicted to show negligible temperature dependence of the deuterium KIE in the 300–600 K range. 

The KIE of the HS^−^ + CH_3_CH_2_Br system, which was slightly normal at 300 K, was predicted to *decrease* at higher temperature. While this may look peculiar, it is understandable by a detailed factor analysis of the vibrational contribution as a function of temperature, as shown in Table S6 in the Supplementary Material. As shown in Table 5, the HS^−^ + CH_3_CH_2_Br reaction has a particularly high deuterium KIE at 300 K which is consistent with the prediction by TST. The high value was due to the high vibrational contribution of 0.831 which was highest among the reactions in the current study. As shown in Table S6, the normal contribution to the KIEs from the mid-frequency modes was higher than other reactions, and it showed stronger negative temperature dependence. This negative dependence of the mid-frequency modes offset the regular positive temperature dependence of the high-frequency modes such that the overall vibrational contribution and the deuterium KIEs showed interesting negative temperature dependence. For reactions with negative barriers, the bottleneck may shift back towards the collision process at lower temperature since the TST rate constants would increase as temperature decrease while the capture rate constant is less sensitive to the temperature. Thus we also include the calculated KIE^CUS^ in Table 6 for comparison.

**Table 6:** Calculated deuterium KIEs and their vibrational contributions at different temperature *a*.

	**ClO^−^ + CH_3_Cl (0.85) *^b^***			**ClO^−^ + CH_3_CH_2_Cl (0.99) *^b^***			**BrO^−^ + CH_3_CH_2_Cl (0.96) *^b^***		
**T(K)**	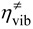	**KIE^TST^**	**KIE^CUS^**	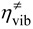	**KIE^TST^**	**KIE^CUS^**	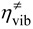	**KIE^TST^**	**KIE^CUS^**
100	0.338	0.571	1.015	0.636	0.875	1.016	0.639	0.896	1.022
200	0.481	0.813	0.936	0.703	0.966	1.007	0.698	0.978	1.005
300	0.527	0.890	0.901	0.717	0.987	0.990	0.710	0.995	0.996
400	0.544	0.919	0.922	0.719	0.989	0.990	0.710	0.995	0.995
500	0.552	0.933	0.934	0.718	0.988	0.988	0.708	0.992	0.992
600	0.557	0.941	0.942	0.718	0.987	0.987	0.707	0.990	0.991
	**Cl^−^ + CH_3_Br (0.88) ^*b*^**			**F^−^(H_2_O) + CH_3_Cl (0.85) ^*b*^**			**HS^−^ + CH_3_CH_2_Br (1.02) ^*b*^**		
**T(K)**	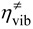	**KIE^TST^**	**KIE^CUS^**	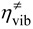	**KIE^TST^**	**KIE^CUS^**	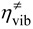	**KIE^TST^**	**KIE^CUS^**
100	0.553	0.691	0.762	0.273	0.470	0.642	0.957	1.235	1.005
200	0.695	0.868	0.871	0.425	0.732	0.735	0.868	1.122	1.066
300	0.732	0.915	0.916	0.481	0.828	0.829	0.831	1.073	1.068
400	0.742	0.928	0.929	0.505	0.870	0.871	0.807	1.042	1.041
500	0.746	0.933	0.934	0.518	0.892	0.893	0.791	1.022	1.022
600	0.749	0.937	0.937	0.527	0.907	0.908	0.782	1.010	1.010

*^a^* In calculating the KIE^CUS^, the energy barriers used were obtained by fitting the *k*^CUS^ to reproduce the experimental rate constants of the unsubstituted systems. The fitted barriers were listed in the supplementary material. *^b^* Values in parentheses are experimental KIEs at ~300 K, see Table 3.

**Figure 1:**
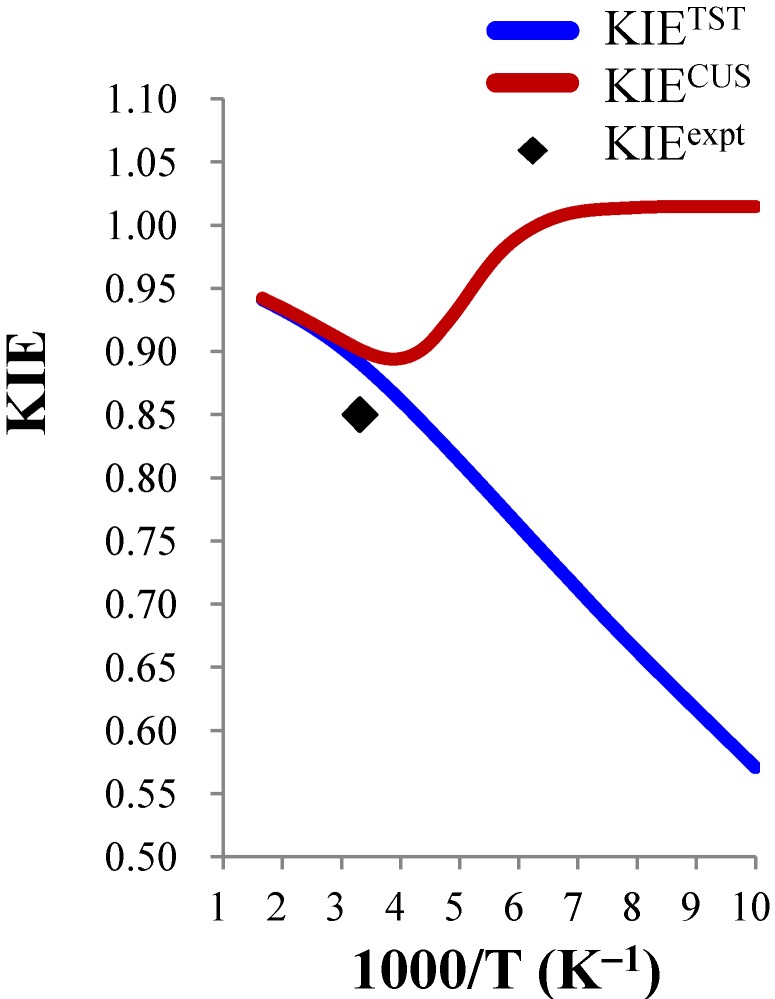
Temperature dependence of the calculated deuterium KIEs of the gas phase ClO^−^ + CH_3_Cl reaction using the TST and CUS methods.

As expected, for the slower reactions, Cl^−^ + CH_3_Br and F^−^(H_2_O) + CH_3_Cl, the agreement between KIE^TST^ and KIE^CUS^is very good down to 200 K. However, for the faster reactions, ClO^−^ + CH_3_Cl and ClO^−^ + CH_3_CH_2_Cl, significant discrepancies occur below 300 K. The comparison of calculated deuterium KIE^TST^ and KIE^CUS^ for the ClO^−^ + CH_3_Cl system from 100 K to 600 K is also shown in Figure 1. The discrepancies are small above 300 K, but are very large at low temperature. Interestingly, a minimum value of KIE^CUS^(0.90) was predicted at about 250 K. Apparently, the CUS theory needs to be used below 300 K to obtain meaningful results.

#### 3.3.4. Tunneling Effects on Rate Constants and KIEs

It has not been established whether tunneling effects can make significant contribution to the rate constants and KIEs of the S_N_2 reactions. On one hand, the reaction paths are dominated by heavy atom movements, and thus the reactions are not very likely to have very strong tunneling effects. On the other hand, the widths of the energy barriers along the reaction paths are small due to the stable ion-dipole complexes before and after the transition states, and the tunneling might be important if there are sizable barriers. Recently there are reports [44,45,46,47,48,49,50] that carbon atoms and oxygen atoms can participate in the tunneling to significant extents. Furthermore, a recent study [25] showed that the carbon atom tunneling could be important in a microsolvated S_N_2 reaction. In the current study the S_N_2 reaction of CN^−^ + CH_3_OCl → OCl^−^ + CH_3_CN was selected to study the tunneling effects. 

**Figure 2:**
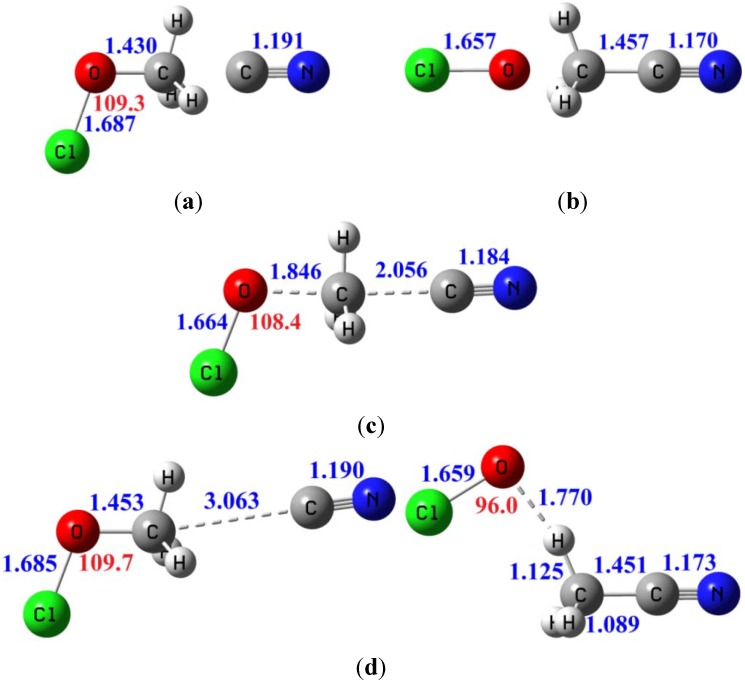
Calculated structures in the gas-phase S_N_2 reaction of CN^−^ + CH_3_OCl at the MP2/aug-cc-pVTZ level. Bond lengths are in Å (blue) and bond angles in degrees (red). (**a**) Reactants (**b**) Products (**c**) Transition state (**d**) Ion-dipole complex.

The calculated energetic is shown in Table 7 and the calculated structures of the reactants, products, TS, and the ion-dipole complexes are shown in Figure 2. The reaction has a sizable barrier height (~10 kcal/mol) and is still significantly exoergic (by ~10 kcal/mol) to keep the barrier width small, and the system is also free from the solvent perturbation.

**Table 7:** Calculated reaction energetic *a* (in kcal/mol) of the gas-phase CN^−^ + CH_3_OCl → OCl^−^ + CH_3_CN reaction.

	**Erxn *^b^***	**ion-dipole complex CN^−^...CH_3_OCl**	**ion-dipole complex OCl^−^...CH_3_CN**	**Barrier height**
B3LYP/6-311+G(d,p)	−11.4	−9.8	−29.6	8.8
MP2/aug-cc-pVDZ	−10.4	−10.0	−29.9	9.8
MP2/aug-cc-pVTZ	−11.9	−9.8	−31.0	10.1
CCSD(T)/aug-cc-pVTZ ^*c*^	−9.9	−9.8	−28.7	9.8

*^a^* All energies relative to CN^−^ + CH_3_OCl; ^*b*^ Energy of reaction; ^*c*^ Using MP2/aug-cc-pVDZ structures.

**Figure 3:**
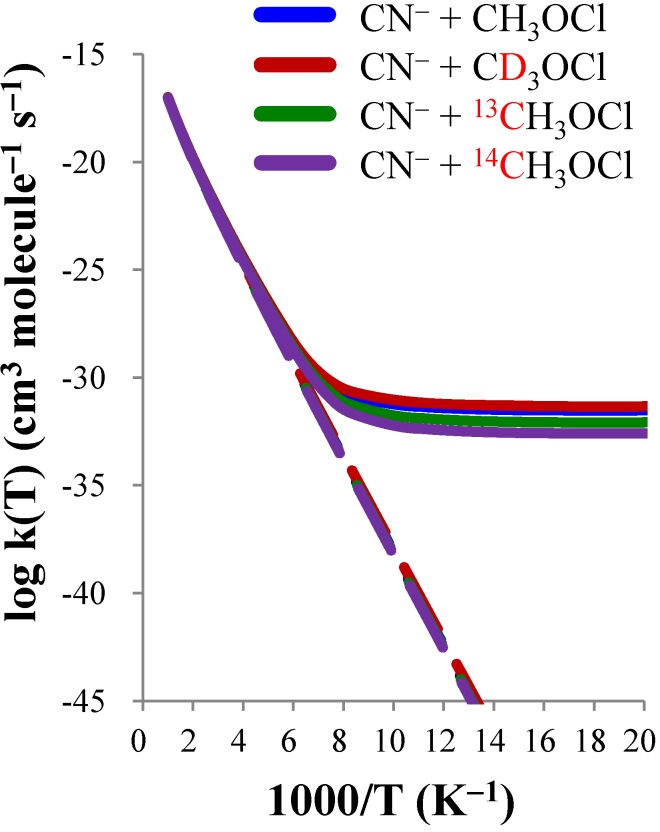
The Arrhenius plots of the calculated rate constants of the gas phase CN^−^ + CH_3_OCl reaction and its three isotopically substituted analogs. The broken and solid lines indicate results obtained at the TST and CVT/SCT levels, respectively.

**Figure 4:**
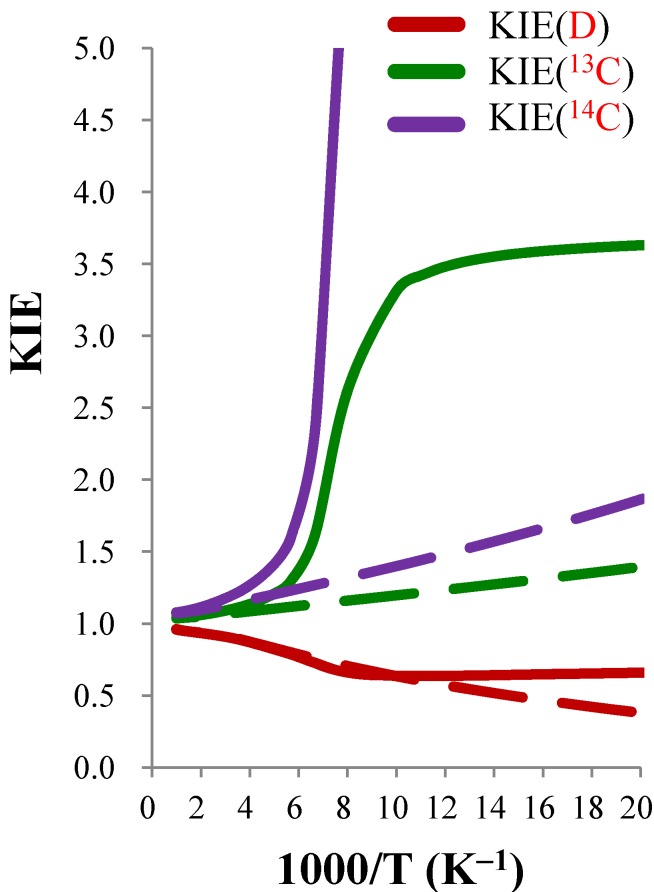
Calculated temperature dependence of the deuterium, ^13^C, and ^14^C KIEs of the gas phase CN^−^ + CH_3_OCl reactions. The broken and solid lines indicated results obtained at the TST and CVT/SCT levels, respectively.

Table 8 shows the calculated TST and CVT/SCT rate constants of CN^−^ + CH_3_OCl (*k*H), CN^−^ + CD_3_OCl (*k*D), CN^−^ + ^13^CH_3_OCl (*k*13C), and CN^−^ + ^14^CH_3_OCl (*k*14C) from 50 to 600 K, and Figure 3 shows the corresponding Arrhenius plots. The apparent curvature in Figure 3 shows that the tunneling effects are indeed important at low temperature. Table 8 shows that even at 300 K the tunneling increases the TST rate constants by approximately 50% for all four reactions. At 250 K tunneling almost doubles all the rate constants, and at 150 K tunneling increases the *k*H and *k*D by a factor of ~20 and increases *k*13C and *k*14C by factors of ~15 and ~10, respectively. Unfortunately the magnitudes of the rate constants calculated in the current reactions were well below the detection thresholds of current experimental techniques. However, the current study serves as a pioneer probe into an experimentally uncharted region where the tunneling plays a very important role in the gas-phase S_N_2 reactions. Table 9 shows the calculated KIEs by TST and CVT/SCT theory where the KIE(D), KIE(^13^C), and KIE(^14^C) are defined as *k*H / *k*D, *k*13C / *k*H, and *k*14C / *k*H, respectively, and the tunneling contributions to the KIE as defined by Equation (22) are also included. It is interesting to notice that while tunneling increase the rate constant significantly below 300 K, the KIE(D) calculated with and without tunneling (CVT/SCT and TST) are almost the same above 100 K. This means the tunneling does not directly involve the motion of hydrogen atoms. At the lowest temperature, the tunneling increases the very inverse KIE(D) predicted by TST because in tunneling dominated reactions, the rate constants are less sensitive to the effective barrier height. On the other hand, the tunneling increase the KIE(^13^C) and KIE(^14^C) by 3% and 5%, respectively at 300 K. The tunneling contributions are significantly normal at lower temperature and increase the KIE(^13^C) and KIE(^14^C) by factors of 2.8 and 7.0 at 100 K, respectively. The plots of the predicted KIEs as functions of temperature are shown in Figure 4. Tunneling effects make dramatic increase in predicted KIE(^13^C) and KIE(^14^C) below 200 K while the KIE(D) was less affected. The current results thus suggest that the tunneling directly involves the motion of the carbon atom. This is consistent with a previous study [25] on the S_N_2 reaction of F^−^(H_2_O) + CH_3_F.

**Table 8:** Calculated rate constants (cm^3^ molecule^−1^s^−1^) by the TST and CVT/SCT methods.

	**CN^−^ + CH_3_OCl**		**CN^−^ + CD_3_OCl**		**CN^−^ + ^13^CH_3_OCl**		**CN^−^ + ^14^CH_3_OCl**	
**T(K)**	**TST**	**CVT/SCT**	**TST**	**CVT/SCT**	**TST**	**CVT/SCT**	**TST**	**CVT/SCT**
50	2.16(−60) ^*a*^	3.01(−32)	5.70(−60)	4.57(−32)	1.55(−60)	8.30(−33)	1.16(−60)	2.54(−33)
100	8.22(−39)	5.82(−32)	1.30(−38)	9.14(−32)	6.87(−39)	1.76(−32)	5.88(−39)	5.94(−33)
150	1.64(−31)	3.32(−30)	2.16(−31)	4.56(−30)	1.44(−31)	2.06(−30)	1.29(−31)	1.42(−30)
200	8.72(−28)	2.79(−27)	1.05(−27)	3.39(−27)	7.90(−28)	2.31(−27)	7.25(−28)	1.97(−27)
250	1.70(−25)	3.30(−25)	1.94(−25)	3.80(−25)	1.56(−25)	2.91(−25)	1.46(−25)	2.61(−25)
300	6.23(−24)	9.65(−24)	6.92(−24)	1.07(−23)	5.81(−24)	8.74(−24)	5.47(−24)	8.03(−24)
400	6.69(−22)	8.40(−22)	7.22(−22)	9.09(−22)	6.33(−22)	7.83(−22)	6.02(−22)	7.36(−22)
500	1.28(−20)	1.47(−20)	1.37(−20)	1.57(−20)	1.22(−20)	1.39(−20)	1.17(−20)	1.32(−20)
600	1.02(−19)	1.11(−19)	1.08(−19)	1.18(−19)	9.73(−20)	1.05(−19)	9.36(−20)	1.01(−19)

^*a*^ 2.16(–60) means 2.16 × 10^−60^.

**Table 9:** Calculated KIEs and its tunneling contributions by the TST and CVT/SCT theory.

	**KIE(D)**			**KIE(^13^C)**			**KIE(^14^C)**		
**T(K)**	**TST**	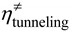	**CVT/SCT**	**TST**	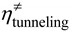	**CVT/SCT**	**TST**	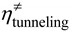	**CVT/SCT**
50	0.380	1.734	0.659	1.394	2.604	3.629	1.863	6.356	11.842
100	0.635	1.003	0.637	1.196	2.768	3.310	1.399	7.003	9.797
150	0.758	0.959	0.727	1.134	1.422	1.613	1.267	1.848	2.342
200	0.831	0.990	0.822	1.103	1.095	1.208	1.203	1.175	1.413
250	0.874	0.995	0.870	1.084	1.047	1.136	1.165	1.087	1.266
300	0.901	0.996	0.897	1.072	1.030	1.104	1.140	1.054	1.201
400	0.926	0.997	0.924	1.057	1.016	1.073	1.110	1.028	1.142
500	0.937	0.997	0.934	1.049	1.010	1.059	1.094	1.018	1.114
600	0.943	0.997	0.940	1.044	1.007	1.051	1.085	1.013	1.099

The KIEs calculated in the current study were based on potential energy surface calculated using the Born-Oppenheimer (BO) approximation which gives identical electronic energies at the same molecular geometry regardless of the isotopic masses. The BO approximation is usually valid for stable molecules on the ground electronic state. Recent studies showed that the corrections to the BO approximation calculated using the diagonal BO correction (DBOC) [96] for small molecules were in the range of 1–3 kcal/mol for total electronic energies, a few cm^–^1 for vibrational frequencies, and less than 0.001 Å for bond lengths [96,97]. Recent theoretical studies with DBOC on the H + H_2_ system [98,99] also showed that the barrier heights changed by 0.1–0.2 kcal/mol on different deuterated reactions. For example, the barrier heights difference calculated by DBOC between the H + H_2_ and D + D_2_ was only 0.08 kcal/mol [98]. Minor differences on the rate constants and deuterium KIEs were observed with and without the DBOC. Since the DBOC tends to increase the barrier height slightly, and the increase is smaller for the deuterated systems, the deuterium KIEs decreased slightly with the DBOC. The recent study by Fleming *et al.* [99] showed the validity of the BO approximation in calculating the KIEs over a dramatic isotopic mass ratio of 36. However, for the S_N_2 reactions we investigated in the current study, the hydrogens or deuteriums were not directly involved in the bond-breaking or bond-forming process. Thus the errors due to the BO approximation in barrier heights are expected to be negligible. The C–H vibrational frequencies of alkyl halides are expected to change by only a few cm^–^^1^ by DBOC, and this would only have very minor effects on the calculated vibrational contribution to the KIEs. The tunneling probability in principle could be affected because the molecular geometry and potential energies along the tunneling paths would also be affected by the non-BO treatment. However for the S_N_2 reactions, the reaction paths involve primarily the movement of heavy atoms, and, as shown above, the most important tunneling contribution is from the central carbon atom. It is thus expected the tunneling contribution to the KIEs would not be significantly affected by the non-BO treatment.

## 4. Conclusions

We have shown in this study that how the kinetic isotope effects (KIEs) of the gas-phase S_N_2 reactions can be realistically modeled. By using the ion-molecular collision theory, the transition state theory (TST), the canonical unified statistical theory (CUS), canonical variational transition state theory with small-curvature (CVT/SCT) tunneling correction, and sometimes the combinations of the above theories, the rate constants and the kinetic isotope effects of the ion-molecule S_N_2 reactions within a very large range of the reaction efficiencies (1.0 to 10^–15^) and barrier heights (−10 to 10 kcal/mol) can be calculated, and the experimental trends can be explained. We have shown that for very fast reactions (with rate constants ~10^9^ cm^3^·molecule^–1^·s^–1^), the bottleneck is located on the ion-molecule collisions, and the reaction would show very small (close to unity) kinetic isotope effects, which is consistent with experimental data. For reactions with small barriers where the accurate TST rate constants are significantly lower than the capture rate constants (or approximately with reaction efficiencies less than 10%), the TST is a good model for the rate constants and KIEs, and modeling the KIEs alone does not even need accurate barrier heights. Our benchmark study showed that commonly used electronic structure methods tend to overestimate the values of the deuterium KIEs, and the MP2/aug-cc-pVDZ performed best in reproducing eleven experimentally measured values. For reactions with intermediate efficiencies or with slightly lower barriers where the accurate TST rate constants are about the same order of magnitude as that of the capture rate constants, the CUS theory, which takes into account both the capture and TST rate constants and makes smooth transition between these two limiting cases, can be applied. However, in such cases accurate modeling of KIEs does require accurate barrier heights. We also showed that for reaction with large barrier heights, the tunneling effects can make significant contributions both to the rate constants and to the carbon KIEs at and below room temperature. This thus suggested that the carbon atom tunneling could be significant in gas-phase S_N_2 reactions.

## Supplementary Materials

Supplementary Materials, including Tables of calculated relative energies, zero-point energies, fitted energy barriers, and KIEs prediction by various theoretical methods can be accessed at: http://www.mdpi.com/1420-3049/18/4/4816/s1.

## References

[1] Wladkiwski B.D., Wilbur J.L., Brauman J.I. (1994). Intrinsic structure-reactivity relationships in gas-phase S_N_2 reactions: Identity exchange of substituted benzyl chlorides with chloride ion. J. Am. Chem. Soc..

[2] Meng Q., Gogoll A., Thibblin A. (1997). Concerted and stepwise solvolytic elimination and substitution reactions: Stereochemistry and substituent effects. J. Am. Chem. Soc..

[3] Gronert S., Fagin A.E., Wong L. (2007). Direct measurements of deuterium kinetic isotope effects in anionic, gas-phase substitution and elimination reactions. J. Am. Chem. Soc..

[4] Poirier R.A., Wang Y., Westaway K.C. (1994). A theoretical study of the relationship between Secondary α-Deuterium kinetic isotope effects and the structure of S_N_2 transition states. J. Am. Chem. Soc..

[5] Graul S.T., Bowers M.T. (1994). Vibrational excitation in products of nucleophilic substitution: The dissociation of metastable X^−^ (CH_3_Y) in the gas phase. J. Am. Chem. Soc..

[6] Cyr D.M., Scarton M.G., Wiberg K.B., Johnson M.A., Nonose S., Hirokawa J., Tanaka H., Kondow T., Morris R.A., Viggiano A.A. (1995). Observation of the XY^−^ abstraction products in the ion-molecule reactions X^−^ + RY → XY^−^ + R: An alternative to the S_N_2 mechanism at suprathermal collision energies. J. Am. Chem. Soc..

[7] Laerdahl J.K., Uggerud E. (2002). Gas phase nucleophilic substitution. Int. J. Mass Spectrom..

[8] Gronert S., Depuy C.H., Bierbaum V.M. (1991). Deuterium isotope effects in gas-phase reactions of alkyl halides: Distinguishing E2 and S_N_2 pathways. J. Am. Chem. Soc..

[9] Almerindo G.I., Pliego R. (2005). Ab Initio Study of the S_N_2 and E2 Mechanisms in the reaction between the cyanide ion and ethyl chloride in dimethyl sulfoxide solution. Org. Lett..

[10] Bento A.P., Solà M., Bickelhaupt F.M. (2008). E2 and S_N_2 Reactions of X^−^ + CH_3_CH_2_X (X = F, Cl); an *ab Initio* and DFT Benchmark Study. J. Chem. Theory Comput..

[11] Matsson O., Dybala-Defratyka A., Rostkowski M., Paneth P., Westaway K.C. (2005). A theoretical investigation of α-Carbon kinetic isotope effects and their relationship to the transition-state structure of S_N_2 reactions. J. Org. Chem..

[12] Fang Y.-R., MacMillar S., Eriksson J., Kołodziejska-Huben M., Dybała-Defratyka A., Paneth P., Matsson O., Westaway K.C. (2006). The effect of solvent on the structure of the transition state for the S_N_2 reaction between cyanide ion and ethyl chloride in DMSO and THF probed with six different kinetic isotope effects. J. Org. Chem..

[13] Hu W.-P., Truhlar D.G. (1996). Factors affecting competitive ion-molecule reactions: ClO^−^ + C_2_H_5_Cl and C_2_D_5_Cl via E2 and S_n_2channels. J. Am. Chem. Soc..

[14] Villano S.M., Kato S., Bierbaum V.M. (2006). Deuterium kinetic isotope effects in gas-phase S_N_2 and E2 reactions: Comparison of experiment and theory. J. Am. Chem. Soc..

[15] Villano S.M., Eyet N., Lineberger W.C., Bierbaum V.M. (2009). Reactions of α-nucleophiles with alkyl chlorides: Competition between S_N_2 and E2 mechanisms and the gas-phase α-effect. J. Am. Chem. Soc..

[16] Garver J.M., Fang Y.-R., Eyet N., Villano S.M., Bierbaum V.M., Westaway K.C. (2010). A Direct comparison of reactivity and mechanism in the gas phase and in solution. J. Am. Chem. Soc..

[17] Zhao X.G., Tucker S.C., Truhlar D.G. (1991). Solvent and secondary kinetic isotope effects for the microhydrated S_N_2 reaction of C1^−^(H_2_O)_n_ with CH_3_Cl. J. Am. Chem. Soc..

[18] Viggiano A.A., Morris R.A., Paschkewitz J.S., Paulson J. (1992). Kinetics of the gas-phase reactions of C1– with CH_3_Br and CD_3_Br: Experimental evidence for nonstatistical behavior?. J. Am. Chem. Soc..

[19] Boyd R.J., Kim C.K., Shi Z., Weinberg N., Wolfe S. (1993). Secondary H/D Isotope effects and transition state looseness in nonidentity methyl transfer reactions. Implications for the concept of enzymatic catalysis via transition state compression. J. Am. Chem. Soc..

[20] O’Hair R.A.J., Dang T.T., DePuy C.H., Bierbaum V.M. (1994). Measurements of solvent and secondary kinetic isotope effects for the gas-phase S_N_2 reactions of fluoride with methyl halides. J. Am. Chem. Soc..

[21] Hu W.-P., Truhlar D.G. (1994). Modeling transition state solvation at the single-molecule level: Test of correlated *ab Initio* predictions against experiment for the gas-phase S_N_2 reaction of microhydrated fluoride with methyl chloride. J. Am. Chem. Soc..

[22] Hu W.-P., Truhlar D.G. (1995). Deuterium kinetic isotope effects and their temperature dependence in the gas-phase S_N_2 reactions X– + CH_3_Y → CH_3_X + Y– (X, Y = C1, Br, I). J. Am. Chem. Soc..

[23] Viggiano A.A., Arnold S.T., Morris R.A., Ahrens A.F., Hierl P.M. (1996). Temperature dependences of the rate constants and branching ratios for the reactions of OH^−^(H_2_O)_0–4_ + CH_3_Br. J. Phys. Chem..

[24] Davico G.E. (2006). Interpretation of the gas-phase solvent deuterium kinetic isotope effects in the S_N_2 reaction mechanism: Comparison of theoretical and experimental results in the reaction of microsolvated fluoride ions with methyl halides. J. Phys. Chem. A.

[25] Chen J.-L., Hu W.-P. (2012). Theoretical study on the gas-phase S_N_2 reaction of microhydrated fluoride with methyl fluoride. J. Chin. Chem. Soc..

[26] Wu Y.-R., Hu W.-P. (1999). Reaction dynamics study on the tunneling effects of a microsolvated E2 reaction: FO^−^(H_2_O) + C_2_H_5_Cl → HOF(H_2_O) + C_2_H_4_ + Cl^−^. J. Am. Chem. Soc..

[27] Eyet N., Villano S.M., Kato S., Bierbaum V.M. (2007). Deuterium kinetic isotope effects in microsolvated gas-phase E2 reactions. J. Am. Soc. Mass Spectrom..

[28] Eyet N., Villano S.M., Bierbaum V.M. (2013). Gas-Phase reactions of microsolvated fluoride ions: An investigation of different solvents. J. Phys. Chem. A.

[29] Pabis A., Paluch P., Szala J., Paneth P. (2009). A DFT study of the kinetic isotope effects on the competing S_N_2 and E2 reactions between hypochlorite anion and ethyl chloride. J. Chem. Theory. Comput..

[30] Eyring H. (1935). The activated complex in chemical reactions. J. Chem. Phys..

[31] Laidler K.J., King M.C. (1983). The development of transition-state theory. J. Phys. Chem..

[32] Truhlar D.G., Hase W.L., Hynes J.T. (1983). Current status of transition-state theory. J. Phys. Chem..

[33] Truhlar D.G., Garrett B.C., Klippenstein S.J. (1996). Current status of transition-state theory. J. Phys. Chem..

[34] Hirschfelder J.O., Wigner E. (1939). Some quantum mechanical considerations in the theory of reactions involving an activation energy. J. Chem. Phys..

[35] Truhlar D.G., Isaacson A.D., Garrett B.C., Baer M. (1985). Generalized Transition State Theory. Theory of Chemical Reaction Dynamics.

[36] Truhlar D.G., Garrett B.C. (1980). Variational transition-state theory. Acc. Chem. Res..

[37] Miller W.H. (1976). Unified statistical model for “complex” and “direct” reaction mechanisms. J. Chem. Phys..

[38] Garrett B.C., Truhlar D.G. (1982). Canonical unified statistical model. Classical mechanical theory and applications to collinear reactions. J. Chem. Phys..

[39] Chesnavich W.J., Su T., Bowers M.T. (1980). Collisions in a noncentral field: A variational and trajectory investigation of ion-dipole capture. J. Chem. Phys..

[40] Celli F., Weddle G., Ridge D.P. (1980). On statistical and thermodynamic approaches to ion polar molecule collisions. J. Chem. Phys..

[41] Garrett B.C., Truhlar D.G., Magnuson A.W. (1982). New semiempirical method of modeling potential energy surfaces for generalized TST and application to the kinetic isotope effects in the Cl–H–H system. J. Chem. Phys..

[42] Lu D.-H., Maurice D., Truhlar D.G. (1990). What is the effect of variational optimization of the transition state on α-Deuterium secondary kinetic isotope effects? A prototype: CD_3_H + H 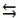 CD_3_ + H_2_. J. Am. Chem. Soc..

[43] Zhao X.G., Lu D.-H., Liu Y.-P., Lynch G.C., Truhlar D.G. (1992). Use of an improved ion-solvent potential-energy function to calculate the reaction rate and α-deuterium and microsolvation kinetic isotope effects for the gas-phase S_N_2 reaction of Cl^−^(H_2_O) with CH_3_Cl. J. Chem. Phys..

[44] Huang C.-H., Tsai L.-C., Hu W.-P. (2001). Dual-Level direct dynamics study on the diels-alder reaction of ethylene and 1,3-Butadiene. J. Phys. Chem. A.

[45] Albu T.V., Lynch B.J., Truhlar D.G., Goren A.C., Hrovat D.A., Borden W.T., Moss R.A. (2002). Dynamics of 1,2-Hydrogen migration in carbenes and ring expansion in cyclopropylcarbenes. J. Phys. Chem. A.

[46] Zuev P.S., Sheridan R.S., Albu T.V., Truhlar D.G., Hrovat D.A., Borden W.T. (2003). Carbon tunneling from a single quantum state. Science.

[47] Datta A., Hrovat D.A., Borden W.T. (2008). Calculations predict rapid tunneling by carbon from the vibrational ground state in the ring opening of cyclopropylcarbinyl radical at cryogenic temperatures. J. Am. Chem. Soc..

[48] Gonzalez-James O.M., Zhang X., Datta A., Hrovat D.A., Borden W.T., Singleton D.A. (2010). Experimental evidence for heavy-atom tunneling in the ring-opening of cyclopropylcarbinyl radical from intramolecular ^12^C/^13^C kinetic isotope effects. J. Am. Chem. Soc..

[49] Chen J-.L., Hu W.-P. (2011). Theoretical Prediction on the Thermal Stability of Cyclic Ozone and Strong Oxygen Tunneling. J. Am. Chem. Soc..

[50] Schreiner P.R., Reisenauer H.P., Ley D., Gerbig D., Wu C.-H., Allen W.D. (2011). Methylhydroxycarbene: Tunneling control of a chemical reaction. Science.

[51] Wigner E. (1932). On the quantum correction for thermodynamic equilibrium. Phys. Rev..

[52] Wigner E. (1932). Über das Überschreiten von Potentialschwellen bei chemischen Reaktionen. Z. Physik. Chem..

[53] Lu D.-H., Truong T.N., Melissas V.S., Lynch G.C., Liu Y.-P., Garrett B.C., Steckler R., Isaacson A.D., Rai S.N., Hancock G.C. (1992). POLYRATE 4: A new version of a computer program for the calculation of chemical reaction rates for polyatomics. Comput. Phys. Commun..

[54] Liu Y.-P., Lynch G.C., Truong T.N., Lu D.-H., Truhlar D.G., Garrett B.C. (1993). Molecular modeling of the kinetic isotope effect for the [1,5] sigmatropic rearrangement of *cis*-1,3-pentadiene. J. Am. Chem. Soc..

[55] Liu Y.-P., Lu D.-H., Gonzalez-Lafont A., Truhlar D.G., Garrett B.C. (1993). Direct dynamics calculation of the kinetic isotope effect for an organic hydrogen-transfer reaction, including corner-cutting tunneling in 21 dimensions. J. Am. Chem. Soc..

[56] Truong T.N., Lu D.-H., Lynch G.C., Liu Y.-P., Melissas V.S., Gonzalez-Lafont A., Rai S.N., Steckler R., Garrett B.C., Joseph T. (1993). MORATE: A program for direct dynamics calculations of chemical reaction rates by semiempirical molecular orbital theory. Comput. Phys. Commun..

[57] Stephens P.J., Devlin F.J., Chabalowski C.F., Frisch M.J. (1994). *Ab Initio* calculation of vibrational absorption and circular dichroism spectra using density functional force fields. J. Phys. Chem..

[58] Zhao Y., Truhlar D.G. (2008). The M06 suite of density functionals for main group thermochemistry, thermochemical kinetics, noncovalent interactions, excited states, and transition elements: two new functionals and systematic testing of four M06-class functionals and 12 other functionals. Theor. Chem. Acc..

[59] Møller C., Plesset M.S. (1934). Note on an approximation treatment for many-electron systems. Phys. Rev..

[60] Hariharan P.C., Pople J.A. (1973). The influence of polarization functions on molecular orbital hydrogenation energies. Theor. Chim. Acta.

[61] Francl M.M., Pietro W.J., Hehre W.J., Binkley J.S., Gordon M.S., DeFrees D.J., Pople J.A. (1982). Self-consistent molecular orbital methods. XXIII. A polarization-type basis set for second-row elements. J. Chem. Phys..

[62] Clark T., Chandrasrkhar J., Spitznagel G.W., Schleyer P.V.R. (1983). Efficient diffuse function-augmented basis sets for anion calculations. III. The 3-21+G basis set for first-row elements, Li–F. J. Comp. Chem..

[63] Krishnan R., Binkley J.S., Seeger R., Pople J.A. (1980). Self-consistent molecular orbital methods. XX. A basis set for correlated wave functions. J. Chem. Phys..

[64] Glukhovtsev M.N., Pross A., McGrath M.P., Radom L. (1995). Extension of Gaussian-2 (G2) theory to bromine- and iodine-containing molecules: Use of effective core potentials. J. Chem. Phys..

[65] Dunning T.H. (1989). Gaussian basis sets for use in correlated molecular calculations. I. The atoms boron through neon and hydrogen. J. Chem. Phys..

[66] Kendall R.A., Dunning T.H., Harrison R.J. (1992). Electron affinities of the first-row atoms revisited. Systematic basis sets and wave functions. J. Chem. Phys..

[67] Woon D.E., Dunning T.H. (1993). Gaussian basis sets for use in correlated molecular calculations. III. The atoms aluminum through argon. J. Chem. Phys..

[68] Pople J.A., HeadGordon M., Raghavachari K. (1987). Quadratic configuration interaction. A general technique for determining electron correlation energies. J. Chem. Phys..

[69] Miller W.H., Handy N.C., Adams J.E. (1980). Reaction path Hamiltonian for polyatomic molecules. J. Chem. Phys..

[70] Minichino C., Barone V. (1994). From concepts to algorithms for the characterization of reaction mechanisms. H_2_CS as a case study. J. Chem. Phys..

[71] Cossi M., Rega N., Scalmani G., Barone V. (2003). Energies, Structures, and Electronic Properties of Molecules in Solution with the C-PCM Solvation Model. J. Comp. Chem..

[72] Barone V. (2005). Anharmonic vibrational properties by a fully automated second-order perturbative approach. J. Chem. Phys..

[73] Peterson K.A., Shepler B.C., Figgen D., Stoll H. (2006). On the spectroscopic and thermochemical properties of ClO, BrO, IO, and their anions. J. Phys. Chem. A.

[74] Dunning T.H., Peterson K.A., Wilson A.K. (2001). Gaussian basis sets for use in correlated molecular calculations. X. The atoms aluminum through argon revisited. J. Chem. Phys..

[75] Frisch M.J., Trucks G.W., Schlegel H.B., Scuseria G.E., Robb M.A., Cheeseman J.R., Scalmani G., Barone V., Mennucci B., Petersson G.A. (2009). Gaussian 09, Revision A.02.

[76] Lide D.R. (2007). CRC Handbook of Chemistry and Physics, Internet Version 2007.

[77] Hu W.-P., Liu Y.-P., Truhlar D.G. (1994). Variational transition-state theory and semiclassical tunneling calculations with interpolated corrections: A new approach to interfacing electronic structure theory and dynamics for organic reactions. J. Chem. Soc. Faraday Trans..

[78] Corchado J.C., Espinosa-Garcia J., Hu W.-P., Rossi I., Truhlar D.G. (1995). Dual-Level Reaction-Path Dynamics (the /// Approach to VTST with Semiclassical Tunneling). Application to OH + NH_3_ → H_2_O + NH_2_. J. Phys. Chem..

[79] Page M., McIver J.W. (1988). On evaluating the reaction path Hamiltonian. J. Chem. Phys..

[80] Page M., Doubleday C., Mclver J.M. (1990). Following steepest descent reaction paths. The use of higher energy derivatives with *ab initio* electronic structure methods. J. Chem. Phys..

[81] Pulay P., Fogarasi G. (1992). Geometry optimization in redundant internal coordinates. J. Chem. Phys..

[82] Jackels C.F., Gu Z., Truhlar D.G. (1995). Reaction-path potential and vibrational frequencies in terms of curvilinear internal coordinates. J. Chem. Phys..

[83] Nguyen K.A., Jackels C.F., Truhlar D.G. (1996). Reaction-path dynamics in curvilinear internal coordinates including torsions. J. Chem. Phys..

[84] Chuang Y.-Y., Truhlar D.G. (1998). Reaction-Path dynamics in redundant internal coordinates. J. Phys. Chem. A.

[85] Huang C.-H., You R.-M., Lian P.-Y., Hu W.-P. (2000). Improved interpolated correction schemes for dual-level direct dynamics calculation. J. Phys. Chem. A.

[86] Corchado J.C., Chunag Y.-Y., Coitino E.L., Truhlar D.G. (1999). Gaussrate, version 8.2.

[87] Chuang Y.-Y., Corchado J.C., Fast P.L., Villa J., Hu W.-P., Liu Y.-P., Lynch G.C., Nguyen K.A., Jackels C.F., Gu M.Z. (1999). Polyrate—Version 8.2.

[88] Zhao Y., González-Garciá N., Truhlar D.G. (2005). Benchmark database of barrier heights for heavy atom transfer, nucleophilic substitution, association, and unimolecular reactions and its use to test theoretical methods. J. Phys. Chem. A.

[89] Chen J.-L., Hong J.-T., Wu K.-J., Hu W.-P. (2009). The MC-DFT approach to the M06-2X, B2K-PLYP, and B2T-PLYP functionals. Chem. Phys. Lett..

[90] Sun Y.-L., Li T.-H., Chen J.-L., Hu W.-P. (2009). Accurate multi-coefficient electronic structure methods MLSE(Cn)-DFT for thermochemical kinetics. Chem. Phys. Lett..

[91] Zhao Y., Truhlar D.G. (2010). Density Functional Calculations of E2 and S_N_2 Reactions: Effects of the Choice of Density Functional, Basis set, and Self-consistent Iterations. J. Chem. Theory. Comput..

[92] Thornton E.R. (1996). Physical organic chemistry. Annu. Rev. Phys. Chem..

[93] Davico G.E. (1999). Distinguishing the S_N_2 and S_N_2’ Mechanisms in the Gas Phase. Org. Lett..

[94] Davico G.E., Bierbaum V.M. (2000). Reactivity and secondary kinetic isotope effects in the S_N_2 reaction mechanism: Dioxygen radical anion and related nucleophilies. J. Am. Chem. Soc..

[95] Meyer M.P. (2012). New applications of isotope effects in the determination of organic reaction mechanism. Adv. Phys. Org. Chem..

[96] Handy N.C., Lee A.M. (1996). The adiabatic approximation. Chem. Phys. Lett..

[97] Harding M.E., Vazquez J., Ruscic B., Wilson A.K., Gauss J., Stanton J.F. (2008). High-accuracy extrapolated *ab initio* thermochemistry. III. Additional improvements and overview. J. Chem. Phys..

[98] Mielke S.L., Schwenke D.W., Schatz G.C., Garrett B.C., Peterson K.A. (2009). Functional representation for the born-oppenheimer diagonal correction and born-huang adiabatic potential energy surfaces for isotopomers of H_3_. J. Phys. Chem. A.

[99] Fleming D.G., Arseneau D.J., Sukhorukov O., Brewer J.H., Mielke S.L., Schatz G.C., Garrett B.C., Peterson K.A., Truhlar D.G. (2011). Kinetic isotope effects for the reactions of muonic helium and muonium with H_2_. Science.

