# Supplementary material for: Theoretical Analysis on the Kinetic Isotope Effects of Bimolecular Nucleophilic Substitution (SN2) Reactions and Their Temperature Dependence
Source: Molecules. 2013 Apr 23;18(4):4816–43. doi: 10.3390/molecules18044816 (PMC6270110; doi:10.3390/molecules18044816)
Supplement: Supplementary file 1 [file molecules-18-04816-s001.pdf]

Supplementary Materials

Table S1. Calculated reaction energetics (in kcal/mol) of the S_N2 and E2 pathways for the ethyl halide systems at the CCSD(T)/aug-cc-pVTZ//MP2/aug-cc-pVDZ level.

	S_N2		E2	
	$\Delta V^{\ddagger}$	ΔE_{rxn}	$\Delta V^{\ddagger}$	ΔE_{rxn}
$ClO^- + CH_3CH_2Cl$	−7.1(−6.8)[a]	−24.4(−23.2)	0.0(−3.5)	−2.8(−5.5)
$BrO^- + CH_3CH_2Cl$	−6.7(−6.5)	−23.8(−22.8)	0.1(−3.5)	−1.1(−4.0)
$HS^- + CH_3CH_2Br$	−2.8(−2.6)	−26.2(−24.4)	9.6(6.0)	−2.7(−6.4)
$CN^- + CH_3CH_2I$	−2.3(−1.9)	−41.3(−38.9)	8.4(5.0)	−7.3(−9.5)

[a] Energy in the parentheses including the zero-point energy.

Table S2. Calculated reaction energetics (in kcal/mol), the xperimental and theoretical rate constants (in cm^3 $molecule^{-1}$ s^{-1}) and KIEs of the S_N2 reactions (with low or small barrier heights) in the gas phase at various levels of theory.

				$ClO^- + CH_3Cl$ / $ClO^- + CD_3Cl^a$					
	$\Delta V^{\neq}$	ΔE_{rxn}	k^{TST}	k^{expt}	η_{trans}	$\eta^{\neq}_{rot}$	$\eta^{\neq}_{vib}$	KIE^{TST}	KIE^{expt}
M06-2X/6-31+G**	−9.1	−29.3	1.55(−06)[f]	2.01(−10)	1.045	1.616	1.009	1.704	0.85 ± 0.01
M06-2X/6-311+G**	−9.2	−28.3	6.05(−07)		1.045	1.616	0.568	0.958	
M06-2X/aug-cc-pVDZ	−8.0	−26.1	5.81(−08)		1.045	1.616	0.510	0.861	
B3LYP/6-31+G**	−10.5	−27.1	1.66(−05)		1.045	1.613	0.638	1.076	
B3LYP/6-311+G**	−10.6	−26.1	2.56(−05)		1.045	1.611	0.669	1.126	
B3LYP/aug-cc-pVDZ	−9.2	−24.1	1.15(−06)		1.045	1.612	0.553	0.932	
MP2/6-31+G**	−5.9	−27.6	2.76(−09)		1.045	1.615	0.542	0.914	
MP2/6-311+G**	−4.8	−27.3	4.26(−10)		1.045	1.569	0.354	0.581	
MP2/aug-cc-pVDZ	−8.5	−26.5	1.34(−07)		1.045	1.617	0.527	0.891	
MP2/aug-cc-pVTZ	−6.7	−23.1	7.00(−09)		1.045	1.615	0.554	0.935	
				$ClO^- + CH_3CH_2Cl$ / $ClO^- + CD_3CD_2Cl^a$					
	$\Delta V^{\neq}$	ΔE_{rxn}	k^{TST}	k^{expt}	η_{trans}	$\eta^{\neq}_{rot}$	$\eta^{\neq}_{vib}$	KIE^{TST}	KIE^{expt}
M06-2X/6-31+G**	−8.6	−30.6	2.10(−08)	2.25(−10)	1.050	1.310	0.679	0.934	0.99 ± 0.01
M06-2X/6-311+G**	−8.9	−29.7	3.27(−08)		1.050	1.310	0.637	0.876	
M06-2X/aug-cc-pVDZ	−7.0	−27.2	1.31(−09)		1.050	1.310	0.632	0.870	
B3LYP/6-31+G**	−9.3	−28.4	4.02(−07)		1.050	1.310	0.785	1.079	
B3LYP/6-311+G**	−9.7	−27.4	8.69(−07)		1.050	1.309	0.801	1.100	
B3LYP/aug-cc-pVDZ	−7.9	−25.2	8.69(−07)		1.050	1.309	0.801	1.100	
MP2/6-31+G**	−5.0	−28.8	1.62(−10)		1.050	1.310	0.747	1.027	
MP2/6-311+G**	−4.4	−28.5	5.10(−11)		1.050	1.310	0.763	1.049	
MP2/aug-cc-pVDZ	−8.0	−27.6	1.58(−08)		1.050	1.310	0.718	0.987	
MP2/aug-cc-pVTZ	−6.0	−24.5	5.54(−10)		1.050	1.308	0.736	1.011	

Table S2. *Cont.*

				$BrO^- + CH_3Cl$ / $BrO^- + CD_3Cl$[a]					
	$\Delta V^{\neq}$	ΔE_{rxn}	k^{TST}	k^{expt}	η_{trans}	$\eta^{\neq}_{rot}$	$\eta^{\neq}_{vib}$	KIE^{TST}	KIE^{expt}
M06-2X/6-31+G**	−8.3	−27.3	1.85(−07)	1.08(−10)	1.059	1.622	0.601	1.033	0.82 ± 0.03
M06-2X/6-311+G**	−7.5	−25.9	3.11(−08)		1.059	1.623	0.563	0.968	
M06-2X/aug-cc-pVDZ	−6.2	−49.7	3.29(−09)		1.059	1.625	0.586	1.008	
B3LYP/6-31+G**	−10.0	−26.2	5.80(−06)		1.059	1.615	0.635	1.086	
B3LYP/6-311+G**	−9.3	−24.8	2.61(−06)		1.059	1.616	0.716	1.226	
B3LYP/aug-cc-pVDZ	−8.0	−23.3	1.57(−07)		1.059	1.619	0.600	1.028	
MP2/6-31+G**	−5.3	−24.8	9.28(−10)		1.059	1.620	0.534	0.916	
MP2/6-311+G**	−3.6	−24.9	6.64(−11)		1.059	1.620	0.555	0.952	
MP2/aug-cc-pVDZ	−8.2	−26.3	9.10(−08)		1.059	1.625	0.520	0.894	
MP2/aug-cc-pVTZ	−6.4	−22.6	5.13(−09)		1.059	1.623	0.548	0.942	
				$BrO^- + CH_3CH_2Cl$ / $BrO^- + CD_3CD_2Cl$[a]					
	$\Delta V^{\neq}$	ΔE_{rxn}	k^{TST}	k^{expt}	η_{trans}	$\eta^{\neq}_{rot}$	$\eta^{\neq}_{vib}$	KIE^{TST}	KIE^{expt}
M06-2X/6-31+G**	−8.7	−29.7	1.70(−08)	1.07(−10)	1.069	1.310	0.688	0.963	0.96 ± 0.03
M06-2X/6-311+G**	−7.0	−27.1	1.17(−09)		1.069	1.311	0.663	0.928	
M06-2X/aug-cc-pVDZ	−5.5	−25.2	5.88(−11)		1.069	1.312	0.611	0.857	
B3LYP/6-31+G**	−9.7	−28.6	4.04(−07)		1.069	1.308	0.764	1.068	
B3LYP/6-311+G**	−8.2	−26.0	7.23(−08)		1.069	1.309	0.769	1.076	
B3LYP/aug-cc-pVDZ	−6.6	−24.4	4.93(−09)		1.069	1.311	0.708	0.992	
MP2/6-31+G**	−5.3	−27.2	1.50(−10)		1.069	1.310	0.733	1.026	
MP2/6-311+G**	−3.1	−26.2	6.22(−12)		1.069	1.311	0.756	1.060	
MP2/aug-cc-pVDZ	−7.9	−27.8	1.33(−08)		1.069	1.312	0.710	0.995	
MP2/aug-cc-pVTZ	−6.0	−24.4	6.20(−10)		1.069	1.310	0.733	1.027	

Table S2. *Cont.*

	$\Delta V^{\neq}$	ΔE_{rxn}	k^{TST}	k^{expt}	η_{trans}	$\eta^{\neq}_{rot}$	$\eta^{\neq}_{vib}$	KIE^{TST}	KIE^{expt}
				$HS^- + CH_3CH_2Br$ / $HS^- + CD_3CD_2Br$ [b]					
M06-2X/6-31+G**	−5.0	−26.7	2.00(−10)	1.95(−10)	1.016	1.272	0.802	1.036	1.02 ± 0.07
M06-2X/6-311+G**	−5.0	−31.2	4.03(−10)		1.016	1.273	0.808	1.044	
M06-2X/aug-cc-pVDZ	−5.7	−31.8	9.56(−10)		1.016	1.273	0.788	1.019	
B3LYP/6-31+G**	−6.6	−25.2	1.78(−08)		1.016	1.273	0.842	1.088	
B3LYP/6-311+G**	−6.8	−29.3	7.79(−08)		1.016	1.275	0.877	1.136	
B3LYP/aug-cc-pVDZ	−6.8	−29.7	7.00(−08)		1.016	1.275	0.848	1.098	
MP2/6-31+G**	−2.4	−32.8	1.45(−11)		1.016	1.272	0.850	1.098	
MP2/6-311+G**	−0.4	−33.9	1.02(−12)		1.016	1.272	0.907	1.171	
MP2/aug-cc-pVDZ	−3.0	−28.8	4.06(−11)		1.016	1.271	0.831	1.073	
MP2/aug-cc-pVTZ	−1.5	−26.3	3.02(−12)		1.016	1.270	0.842	1.087	
				$Cl^- + CH_3I$ / $Cl^- + CD_3I$ [b]					
	$\Delta V^{\neq}$	ΔE_{rxn}	k^{TST}	k^{expt}	η_{trans}	$\eta^{\neq}_{rot}$	$\eta^{\neq}_{vib}$	KIE^{TST}	KIE^{expt}
M06-2X/6-31+G**	−7.2	−15.9	4.88(−07)	1.66(−10)	1.006	1.230	0.758	0.937	0.84 ± 0.02
M06-2X/6-311+G**	−6.9	−16.3	3.21(−07)		1.006	1.230	0.765	0.947	
M06-2X/aug-cc-pVDZ	−8.1	−17.7	2.13(−06)		1.006	1.230	0.739	0.915	
B3LYP/6-31+G**	−8.7	−13.7	7.33(−06)		1.006	1.229	0.775	0.958	
B3LYP/6-311+G**	−8.9	−14.4	1.28(−05)		1.006	1.229	0.795	0.984	
B3LYP/aug-cc-pVDZ	−9.3	−15.4	1.82(−05)		1.006	1.229	0.762	0.943	
MP2/6-31+G**	−5.3	−20.9	1.65(−08)		1.006	1.229	0.733	0.906	
MP2/6-311+G**	−2.8	−19.1	3.17(−10)		1.006	1.231	0.764	0.946	
MP2/aug-cc-pVDZ	−4.2	−11.9	2.32(−09)		1.006	1.231	0.717	0.889	
MP2/aug-cc-pVTZ	−3.0	−10.5	3.10(−10)		1.006	1.231	0.738	0.914	
CCSD(T)/aug-cc-pVTZ	−4.1	−10.7	2.71(−09)		1.006	1.231	0.758	0.939	

Table S2. *Cont.*

	$Br^- + CH_3I$ / $Br^- + CD_3I$[b]								
	$\Delta V^{\neq}$	ΔE_{rxn}	k^{TST}	k^{expt}	η_{trans}	$\eta^{\neq}_{rot}$	$\eta^{\neq}_{vib}$	KIE^{TST}	KIE^{expt}
M06-2X/6-31+G**	−7.9	−10.4	1.43(−06)	2.89(−11)	1.011	1.240	0.756	0.949	0.76 ± 0.03
M06-2X/6-311+G**	−4.2	−7.5	4.06(−09)		1.011	1.241	0.769	0.965	
M06-2X/aug-cc-pVDZ	−5.2	−7.2	1.93(−08)		1.011	1.240	0.740	0.928	
B3LYP/6-31+G**	−9.1	−9.3	1.44(−05)		1.011	1.239	0.778	0.975	
B3LYP/6-311+G**	−6.4	−7.1	2.55(−07)		1.011	1.239	0.811	1.016	
B3LYP/aug-cc-pVDZ	−6.8	−6.9	3.26(−07)		1.011	1.239	0.765	0.959	
MP2/6-31+G**	−2.3	−11.2	1.11(−10)		1.011	1.240	0.741	0.929	
MP2/6-311+G**	0.1	−9.2	3.74(−12)		1.011	1.242	0.797	1.001	
MP2/aug-cc-pVDZ	−2.5	−5.2	1.71(−10)		1.011	1.241	0.731	0.918	
MP2/aug-cc-pVDZ an[g]	−2.5	−5.2	1.72(−10)		1.011	1.241	0.751	0.943	
MP2/aug-cc-pVDZ an vibrot[h]	−2.5	−5.2	1.67(−10)		1.011	1.241	0.723	0.907	
MP2/aug-cc-pVTZ	−1.8	−4.6	6.01(−11)		1.011	1.241	0.760	0.954	
	$CN^- + CH_3I$ / $CN^- + CD_3I$[c]								
	$\Delta V^{\neq}$	ΔE_{rxn}	k^{TST}	k^{expt}	η_{trans}	$\eta^{\neq}_{rot}$	$\eta^{\neq}_{vib}$	KIE^{TST}	KIE^{expt}
M06-2X/6-31+G**	−8.7	−52.9	3.20(−07)	1.28(−10)	1.005	1.228	0.767	0.946	0.84 ± 0.03
M06-2X/6-311+G**	−8.1	−52.5	1.93(−07)		1.005	1.228	0.753	0.928	
M06-2X/aug-cc-pVDZ	−8.7	−52.6	5.84(−07)		1.005	1.228	0.739	0.912	
B3LYP/6-31+G**	−9.4	−51.2	1.25(−06)		1.005	1.227	0.771	0.950	
B3LYP/6-311+G**	−9.2	−51.9	1.35(−06)		1.005	1.227	0.788	0.972	
B3LYP/aug-cc-pVDZ	−9.6	−51.7	2.38(−06)		1.005	1.227	0.758	0.934	
MP2/6-31+G**	−6.4	−55.6	1.36(−08)		1.005	1.227	0.728	0.898	
MP2/6-311+G**	−3.7	−51.7	4.63(−10)		1.005	1.229	0.747	0.923	
MP2/aug-cc-pVDZ	−6.4	−48.8	4.39(−09)		1.005	1.229	0.713	0.881	
MP2/aug-cc-pVTZ	−4.6	−45.6	2.32(−10)		1.005	1.229	0.715	0.883	
CCSD(T)/aug-cc-pVTZ	−5.4	−43.7	1.04(−09)		1.005	1.228	0.731	0.902	

Table S2. *Cont.*

	$\Delta V^{\neq}$	ΔE_{rxn}	k^{TST}	k^{expt}	η_{trans}	$\eta^{\neq}_{rot}$	$\eta^{\neq}_{vib}$	KIE^{TST}	KIE^{expt}
				$CN^{-} + CH_3CH_2I$ / $CN^{-} + CD_3CD_2I^{c}$					
M06-2X/6-31+G**	−6.0	−51.5	2.34(−09)	2.99(−11)	1.007	1.265	0.742	0.944	0.89 ± 0.02
M06-2X/6-311+G**	−5.6	−51.3	8.04(−10)		1.007	1.266	0.715	0.911	
M06-2X/aug-cc-pVDZ	−6.1	−51.0	5.50(−09)		1.007	1.262	0.717	0.911	
B3LYP/6-31+G**	−6.1	−49.9	5.49(−09)		1.007	1.270	0.769	0.983	
B3LYP/6-311+G**	−6.2	−50.6	7.44(−09)		1.007	1.270	0.784	1.003	
B3LYP/aug-cc-pVDZ	−6.4	−50.3	1.27(−08)		1.007	1.270	0.761	0.973	
MP2/6-31+G**	−2.6	−53.8	3.11(−11)		1.007	1.264	0.739	0.941	
MP2/6-311+G**	−0.4	−49.9	9.28(−13)		1.007	1.264	0.765	0.974	
MP2/aug-cc-pVDZ	−2.8	−45.9	1.52(−11)		1.007	1.264	0.724	0.921	
MP2/aug-cc-pVTZ	−0.9	−42.9	5.48(−13)		1.007	1.263	0.727	0.924	
				$Cl^{-} + CH_3Br$ / $Cl^{-} + CD_3Br^{d}$					
	$\Delta V^{\neq}$	ΔE_{rxn}	k^{TST}	k^{expt}	η_{trans}	$\eta^{\neq}_{rot}$	$\eta^{\neq}_{vib}$	KIE^{TST}	KIE^{expt}
M06-2X/6-31+G**	−4.6	−5.6	3.79(−09)	2.37(−11)	1.013	1.234	0.752	0.940	0.88 ± 0.45
M06-2X/6-311+G**	−3.7	−8.8	1.53(−09)		1.013	1.233	0.764	0.955	
M06-2X/aug-cc-pVDZ	−5.0	−10.5	1.25(−08)		1.013	1.233	0.744	0.929	
B3LYP/6-31+G**	−6.2	−4.4	8.49(−08)		1.013	1.232	0.783	0.977	
B3LYP/6-311+G**	−5.7	−7.3	6.90(−08)		1.013	1.232	0.814	1.016	
B3LYP/aug-cc-pVDZ	−6.3	−8.5	1.52(−07)		1.013	1.232	0.774	0.966	
MP2/6-31+G**	−1.8	−9.7	3.68(−11)		1.013	1.233	0.749	0.936	
MP2/6-311+G**	1.9	−9.9	1.53(−13)		1.013	1.235	0.782	0.978	
MP2/aug-cc-pVDZ	−1.5	−6.7	3.22(−11)		1.013	1.234	0.732	0.915	
MP2/aug-cc-pVDZ an[g]	−1.5	−6.7	3.33(−11)		1.013	1.234	0.766	0.957	
MP2/aug-cc-pVDZ an vibrot[h]	−1.5	−6.7	3.28(−11)		1.013	1.234	0.738	0.923	
MP2/aug-cc-pVTZ	−0.7	−5.9	9.37(−12)		1.013	1.234	0.770	0.963	
CCSD(T)/aug-cc-pVTZ	−2.1	−6.2	4.93(−11)		1.013	1.233	0.775	0.967	

Table S2. *Cont.*

	$CH_3Cl + F^-(H_2O)$ / $CD_3Cl + F^-(H_2O)$[e]								
	$\Delta V^{\ne}$	ΔE_{rxn}	k^{TST}	k^{expt}	η_{trans}	$\eta^{\ne}_{rot}$	$\eta^{\ne}_{vib}$	KIE^{TST}	KIE^{expt}
M06-2X/6-31+G**	−3.3	−23.3	3.87(−12)	1.49(−11)	1.037	1.652	0.477	0.818	0.85 ± 0.03
M06-2X/6-311+G**	−4.4	−23.5	5.51(−11)		1.037	1.328	0.593	0.817	
M06-2X/aug-cc-pVDZ	−3.6	−22.5	1.15(−12)		1.037	1.668	0.456	0.790	
B3LYP/6-31+G**	−6.2	−20.2	1.16(−08)		1.037	1.631	0.518	0.876	
B3LYP/6-311+G**	−7.2	−20.4	1.25(−07)		1.037	1.531	0.591	0.938	
B3LYP/aug-cc-pVDZ	−5.9	−19.4	4.68(−09)		1.037	1.641	0.491	0.835	
MP2/6-31+G**	1.2	−15.2	8.82(−14)		1.037	1.618	0.502	0.842	
MP2/6-311+G**	2.9	−15.3	8.41(−15)		1.037	1.554	0.531	0.856	
MP2/aug-cc-pVDZ	−2.7	−17.5	7.01(−12)		1.037	1.660	0.481	0.829	
MP2/aug-cc-pVTZ	−0.5	−17.0	3.63(−13)		1.037	1.655	0.513	0.882	

[a] Experimental values from ref. 15 at 302 K; [b] Experimental values from ref. 8, calculations done at 300 K; [c] Experimental values from ref. 16 at 298 K; [d] Experimental values from ref. 18 at 300 K; [e] Experimental values from ref. 20 at 302 K; [f] 1.55(−06) means 1.55×10^{-6}; [g] Anharmonic frequencies were calculated at the MP2/aug-cc-pVDZ level; [h] Anharmonic vibrational-rotational couplings were calculated at the MP2/aug-cc-pVDZ level.

Table S3. Calculated reaction energetics (in kcal/mol), and a comparison of experimental and theoretical rate constants (in cm^3 $molecule^{-1}$ s^{-1}) of the CN^- + CH_3I, CN^- + CH_3CH_2I, CN^- + $(CH_3)_2CHI$, and CN^- + $(CH_3)_3CI$ reactions.[a]

	$\Delta V^{\ddagger}$	ΔE_{rxn}	k^{TST}	k^{cap}	k^{expt}	Efficiency	η_{trans}	$\eta^{\neq}_{rot}$	$\eta^{\neq}_{vib}$	KIE^{TST}	KIE^{expt}
CN^- + CH_3I / CN^- + CD_3I											
M06-2X/6-31+G**	−8.7	−52.9	3.20(−07)[b]	2.44(−09)	1.28(−10)	0.052	1.005	1.228	0.767	0.946	0.84 ± 0.03
M06-2X/6-311+G**	−8.1	−52.5	1.93(−07)				1.005	1.228	0.753	0.928	
M06-2X/aug-cc-pVDZ	−8.7	−52.6	5.84(−07)				1.005	1.228	0.739	0.912	
B3LYP/6-31+G**	−9.4	−51.2	1.25(−06)				1.005	1.227	0.771	0.950	
B3LYP/6-311+G**	−9.2	−51.9	1.35(−06)				1.005	1.227	0.788	0.972	
B3LYP/aug-cc-pVDZ	−9.6	−51.7	2.38(−06)				1.005	1.227	0.758	0.934	
MP2/6-31+G**	−6.4	−55.6	1.36(−08)				1.005	1.227	0.728	0.898	
MP2/6-311+G**	−3.7	−51.7	4.63(−10)				1.005	1.229	0.747	0.923	
MP2/aug-cc-pVDZ	−6.4	−48.8	4.39(−09)				1.005	1.229	0.713	0.881	
MP2/aug-cc-pVTZ	−4.6	−45.6	2.32(−10)				1.005	1.229	0.715	0.883	
CCSD(T)/aug-cc-pVTZ	−5.4	−43.7	1.04(−09)				1.005	1.228	0.731	0.902	
CN^- + CH_3CH_2I / CN^- + CD_3CD_2I											
	$\Delta V^{\ddagger}$	ΔE_{rxn}	k^{TST}	k^{cap}	k^{expt}	Efficiency	η_{trans}	$\eta^{\neq}_{rot}$	$\eta^{\neq}_{vib}$	KIE^{TST}	KIE^{expt}
M06-2X/6-31+G**	−6.0	−51.5	2.34(−09)	2.81(−09)	2.99(−11)	0.011	1.007	1.265	0.742	0.944	0.89 ± 0.02
M06-2X/6-311+G**	−5.6	−51.3	8.04(−10)				1.007	1.266	0.715	0.911	
M06-2X/aug-cc-pVDZ	−6.1	−51.0	5.50(−09)				1.007	1.262	0.717	0.911	
B3LYP/6-31+G**	−6.1	−49.9	5.49(−09)				1.007	1.270	0.769	0.983	
B3LYP/6-311+G**	−6.2	−50.6	7.44(−09)				1.007	1.270	0.784	1.003	
B3LYP/aug-cc-pVDZ	−6.4	−50.3	1.27(−08)				1.007	1.270	0.761	0.973	
MP2/6-31+G**	−2.6	−53.8	3.11(−11)				1.007	1.264	0.739	0.941	
MP2/6-311+G**	−0.4	−49.9	9.28(−13)				1.007	1.264	0.765	0.974	
MP2/aug-cc-pVDZ	−2.8	−45.9	1.52(−11)				1.007	1.264	0.724	0.921	
MP2/aug-cc-pVTZ	−0.9	−42.9	5.48(−13)				1.007	1.263	0.727	0.924	

Table S3. *Cont.*

	$CN^- + (CH_3)_2CHI$ / $CN^- + (CD_3)_2CDI$										
	$\Delta V^{\neq}$	ΔE_{rxn}	k^{TST}	k^{cap}	k^{expt}	Efficiency	η_{trans}	$\eta^{\neq}_{rot}$	$\eta^{\neq}_{vib}$	KIE^{TST}	KIE^{expt}
M06-2X/6-31+G**	−2.9	−50.2	2.50(−11)	2.90(−09)	<1.00(−12)	<0.0003	1.008	1.170	0.869	1.024	–
M06-2X/6-311+G**	−2.8	−50.3	2.21(−11)				1.008	1.170	0.903	1.065	
M06-2X/aug-cc-pVDZ	−3.2	−49.8	7.95(−11)				1.008	1.168	0.846	0.996	
B3LYP/6-31+G**	−2.9	−49.1	4.53(−11)				1.008	1.172	0.872	1.030	
B3LYP/6-311+G**	−3.3	−49.9	8.32(−11)				1.008	1.172	0.880	1.039	
B3LYP/aug-cc-pVDZ	−3.4	−49.4	1.15(−10)				1.008	1.172	0.850	1.005	
MP2/6-31+G**	1.5	−52.5	2.01(−14)				1.008	1.169	0.813	0.957	
MP2/6-311+G**	3.3	−48.8	8.12(−16)				1.008	1.169	0.832	0.980	
MP2/aug-cc-pVDZ	1.4	−43.5	1.86(−14)				1.008	1.170	0.787	0.928	
MP2/aug-cc-pVTZ	3.5	−40.5	4.51(−16)				1.008	1.169	0.800	0.943	
	$CN^- + (CH_3)_3CI$ / $CN^- + (CD_3)_3CI$										
	$\Delta V^{\neq}$	ΔE_{rxn}	k^{TST}	k^{cap}	k^{expt}	Efficiency	η_{trans}	$\eta^{\neq}_{rot}$	$\eta^{\neq}_{vib}$	KIE^{TST}	KIE^{expt}
M06-2X/6-31+G**	4.1	−49.6	1.80(−14)	3.13(−09)	1.10(−11)	0.004	1.009	1.133	1.146	1.310	>8
M06-2X/6-311+G**	3.4	−49.9	7.52(−14)				1.009	1.133	1.236	1.413	
M06-2X/aug-cc-pVDZ	3.6	−49.2	3.87(−14)				1.009	1.134	1.131	1.293	
B3LYP/6-31+G**	3.9	−48.8	6.30(−14)				1.009	1.131	1.165	1.329	
B3LYP/6-311+G**	3.3	−49.5	2.41(−13)				1.009	1.131	1.186	1.353	
B3LYP/aug-cc-pVDZ	3.5	−49.0	1.53(−13)				1.009	1.131	1.160	1.324	
MP2/6-31+G**	11.9	−51.6	1.06(−18)				1.009	1.134	1.268	1.450	
MP2/6-311+G**	14.5	−48.2	3.59(−21)				1.009	1.134	1.147	1.313	
MP2/aug-cc-pVDZ	13.9	−41.6	2.42(−23)				1.009	1.129	0.767	0.873	
MP2/aug-cc-pVTZ	15.9	−38.5	4.14(−26)				1.009	1.129	1.059	1.206	

[a] Experimental values from ref. 16 at 298 K.

[b] 3.02(–07) means 3.02 × 10^{-7}

Table S4. Fitted barrier heights (kcal/mol) for Table 6 at the experimental temperature.

	**barrier height**
$ClO^- + CH_3Cl$	–4.6
$ClO^- + CH_3CH_2Cl$	–5.5
$BrO^- + CH_3CH_2Cl$	–5.0
$Cl^- + CH_3Br$	–1.3
$F^-(H_2O) + CH_3Cl$	–3.2
$HS^- + CH_3CH_2Br$	–4.0

Table S5. Calculated energies (in kcal/mol) of eleven gas-phase S_N2 reactions at the MP2/aug-cc-pVDZ level.

	$\Delta V^{\ddagger}$	ΔE_{rxn}	ion-dipole complex		zero-point energy	
			reactant-side	product-side	reactants	transition state
$ClO^{-} + CH_3Cl$	−8.5	−26.5	−13.5	−37.3	24.9	25.5
$ClO^{-} + CH_3CH_2Cl$	−8.0	−27.6	−15.2	−39.5	43.1	43.4
$BrO^{-} + CH_3Cl$	−8.2	−26.3	−13.6	−36.7	24.9	25.4
$BrO^{-} + CH_3CH_2Cl$	−7.9	−27.8	−15.4	−39.3	43.0	43.2
$HS^{-} + CH_3CH_2Br$	−3.0	−28.8	−12.3	−38.6	45.5	45.8
$Cl^{-} + CH_3I$	−4.2	−11.9	−11.7	−21.7	23.1	23.2
$Br^{-} + CH_3I$	−2.5	−5.2	−11.4	−15.5	23.1	23.0
$CN^{-} + CH_3I$	−6.4	−48.8	−10.8	−61.4	25.8	26.4
$CN^{-} + CH_3CH_2I$	−2.8	−45.9	−13.4	−60.3	44.1	44.6
$Cl^{-} + CH_3Br$	−1.5	−6.7	−11.6	−17.4	23.5	23.4
$F^{-}(H_2O) + CH_3Cl$	−2.7	−17.5	−13.5	−27.5	38.0	39.4

Table S6. Factor analysis of $\eta^{\neq}_{vib}$ ($\eta^{\neq}_{vib} = \eta^{\neq}_{vib,low}\ \eta^{\neq}_{vib,mid}\ \eta^{\neq}_{vib,high}$)[a] for the $ClO^{-} + CH_3CH_2Cl$, $BrO^{-} + CH_3CH_2Cl$, and $HS^{-} + CH_3CH_2Br$ reactions at different temperature.

	**$ClO^{-} + CH_3CH_2Cl$**				**$BrO^{-} + CH_3CH_2Cl$**				**$HS^{-} + CH_3CH_2Br$**			
T(K)	$\eta^{\neq}_{vib}$	$\eta^{\neq}_{vib,low}$	$\eta^{\neq}_{vib,mid}$	$\eta^{\neq}_{vib,high}$	$\eta^{\neq}_{vib}$	$\eta^{\neq}_{vib,low}$	$\eta^{\neq}_{vib,mid}$	$\eta^{\neq}_{vib,high}$	$\eta^{\neq}_{vib}$	$\eta^{\neq}_{vib,low}$	$\eta^{\neq}_{vib,mid}$	$\eta^{\neq}_{vib,high}$
100	0.636	0.733	1.401	0.620	0.639	0.724	1.414	0.625	0.957	0.819	2.150	0.543
200	0.703	0.754	1.183	0.787	0.698	0.743	1.188	0.790	0.868	0.806	1.463	0.737
300	0.717	0.759	1.109	0.853	0.710	0.746	1.112	0.855	0.831	0.803	1.269	0.816
400	0.719	0.760	1.066	0.887	0.710	0.748	1.068	0.889	0.807	0.801	1.172	0.859
500	0.718	0.761	1.038	0.909	0.708	0.748	1.039	0.911	0.791	0.801	1.116	0.886
600	0.718	0.761	1.020	0.925	0.707	0.748	1.020	0.926	0.782	0.801	1.080	0.905

[a] "low" denotes contributions from mode with $\nu_i < 500\ cm^{-1}$; "high" denotes contributions from mode with $\nu_i > 2000\ cm^{-1}$; and "mid" denotes the remaining contributions from the middle frequencies.
